# *In vivo* single-molecule imaging of syntaxin1A reveals polyphosphoinositide- and activity-dependent trapping in presynaptic nanoclusters

**DOI:** 10.1038/ncomms13660

**Published:** 2016-12-16

**Authors:** Adekunle T. Bademosi, Elsa Lauwers, Pranesh Padmanabhan, Lorenzo Odierna, Ye Jin Chai, Andreas Papadopulos, Geoffrey J. Goodhill, Patrik Verstreken, Bruno van Swinderen, Frédéric A. Meunier

**Affiliations:** 1 Clem Jones Centre for Ageing Dementia Research, Queensland Brain Institute, The University of Queensland, Brisbane, Queensland 4072, Australia; 2 VIB Center for the Biology of Disease, 3000 Leuven, Belgium; 3 KU Leuven Department of Human Genetics, Leuven Institute for Neurodegenerative Disease (LIND), 3000 Leuven, Belgium; 4 Queensland Brain Institute, The University of Queensland, Brisbane, Queensland 4072, Australia; 5 School of Biomedical Sciences, The University of Queensland, Brisbane, Queensland 4072, Australia; 6 School of Mathematics and Physics, The University of Queensland, Brisbane, Queensland 4072, Australia

## Abstract

Syntaxin1A is organized in nanoclusters that are critical for the docking and priming of secretory vesicles from neurosecretory cells. Whether and how these nanoclusters are affected by neurotransmitter release in nerve terminals from a living organism is unknown. Here we imaged photoconvertible syntaxin1A-mEos2 in the motor nerve terminal of *Drosophila* larvae by single-particle tracking photoactivation localization microscopy. Opto- and thermo-genetic neuronal stimulation increased syntaxin1A-mEos2 mobility, and reduced the size and molecular density of nanoclusters, suggesting an activity-dependent release of syntaxin1A from the confinement of nanoclusters. Syntaxin1A mobility was increased by mutating its polyphosphoinositide-binding site or preventing SNARE complex assembly via co-expression of tetanus toxin light chain. In contrast, syntaxin1A mobility was reduced by preventing SNARE complex disassembly. Our data demonstrate that polyphosphoinositide favours syntaxin1A trapping, and show that SNARE complex disassembly leads to syntaxin1A dissociation from nanoclusters. Lateral diffusion and trapping of syntaxin1A in nanoclusters therefore dynamically regulate neurotransmitter release.

## Abstract (web summary)

Syntaxin1A (Sx1A) is organized in nanoclusters in neurosecretory cells but how these nanoclusters are affected by neurotransmitter release in a living organism is unknown. Here the authors perform single molecule imaging analysis in live fly larvae and show that the lateral diffusion and trapping of Sx1A in nanoclusters are altered by synaptic activity.

The exocytic fusion of neurosecretory vesicles is a key step required for communication between neurons. Different proteins mediate this process, in particular the soluble N-ethylmaleimide sensitive-factor attachment receptor proteins (SNAREs): VAMP2, SNAP-25 and syntaxin1A (Sx1A)^1^. The role of SNARE proteins in mediating synaptic vesicle fusion has been revealed through the use of clostridial neurotoxins that specifically cleave the different SNARE components^2^,^3^. The SNARE proteins assemble before vesicle fusion to form a stable SNARE complex, which comprises four 60–70 amino-acid-long SNARE helical structures. Sx1A contributes one SNARE motif, SNAP-25 inserts two SNARE motifs and VAMP2, the vesicle-bound SNARE, provides the fourth SNARE motif^4^,^5^,^6^. Formation of the SNARE complex is a crucial step in the fusion of the vesicle membrane with the presynaptic plasma membrane and the formation of the fusion pore^7^. Following zippering of the SNARE complex and fusion of the secretory vesicles with the plasma membrane, N-ethylmaleimide-sensitive factor (NSF) and NSF attachment protein alpha (α-SNAP) promote the disassembly of the SNARE complex^8^.

Sx1A is composed of a C-terminal transmembrane domain, a SNARE motif-forming H3 domain and an N-terminal Habc domain^9^. Sx1A mediates the tethering of neurotransmitter-filled vesicles to the presynaptic membrane (docking)^10^ and the priming of the synaptic and neurosecretory vesicles in preparation for calcium ion influx-dependent fusion^8^.

Transmembrane proteins were initially viewed as being freely mobile and dispersed on the presynaptic membrane based on the Singer and Nicolson models of protein diffusion^11^,^12^. However, recent advances in microscopy have revealed Sx1A dynamics on the plasma membrane *in vitro*, with the mobility of the protein ranging from almost immobile to unrestricted^13^. Using direct stochastic optical reconstruction microscopy, immobile Sx1A was shown to form 50–100 nm sized clusters containing about 50–70 individual molecules, and it was proposed that a dynamic exchange equilibrium exists between the clustered and mobile Sx1A (refs 13, 14, 15, 16). Within these nanoclusters, Sx1A molecules were observed to form a density gradient, with more Sx1A at the centre and less at the periphery^13^,^16^. The clustered Sx1A molecules were proposed to mediate exocytosis by acting as docking and priming platforms^16^,^17^,^18^.

The Sx1A nanocluster arrangement is mediated by a variety of factors such as weak electrostatic interactions with specific phospholipids, protein–protein homotypic interactions and hydrophobic interactions between the plasma membrane and the transmembrane portion of Sx1A molecules^19^. The interaction between SNARE motifs of different Sx1A molecules has been found to control the recruitment into clusters^19^. Cholesterol has also been shown to control clustering of Sx1A (refs 20, 21). Electrostatic interactions between the positively charged Sx1A transmembrane domain and negatively charged phosphoinositides such as phosphatidylinositol-4,5-bisphosphate (PtdIns(4,5)*P*_2_) and phosphatidylinositol-3,4,5-trisphosphate (PtdIns(3,4,5)*P*_3_) also modulate Sx1A clustering in neurosecretory cells and in fixed *Drosophila* tissue^22^,^23^,^24^. Furthermore, in *Drosophila* larvae the size of the nanoclusters has recently been shown to vary according to their proximity to the active zone^25^. A mismatch between the length of the transmembrane domain and the lipid bilayer can also induce the clustering of Sx1A (ref. 26). Single-molecule imaging analysis of Sx1A expressed in spinal cord neurons revealed that Sx1A mobility is more restricted at synapses when compared with extrasynaptic sites^27^,^28^.

Our current understanding of Sx1A nanocluster organization and function has been limited by the use of fixed membrane sheets and cells^13^,^15^,^16^,^24^, a recent study has started to shape a more dynamic view of the nanocluster organization in live neurosecretory cells^29^. However, whether and how Sx1A molecules are organized in nanoclusters in presynapses at the level of a live organism is unknown. Indeed, the mechanisms controlling the lateral diffusion and trapping of Sx1A molecules in nanoclusters in the plane of the presynaptic plasma membrane of live nerve terminals remains to be established. More importantly, it is critical to gain a better understanding of whether/how acute synaptic activity might alter Sx1A lateral diffusion and trapping in nanoclusters during stimulation of neurotransmitter release.

Single-particle tracking photoactivation localization microscopy (sptPALM) allows the investigation of the behaviour of single molecules *in vitro* or *in vivo*^30^. We constitutively expressed fluorescently tagged Sx1A (Sx1A-mEos2) in *Drosophila melanogaster* using the endogenous Sx1A promoter, and used slightly oblique illumination to image the surface of the labelled motor nerve terminals in third instar larvae. Our results confirmed that Sx1A is organized in nanoclusters on the presynaptic membrane. Increasing synaptic activity using either optogenetic or thermogenetic stimulation revealed an activity-dependent increase in Sx1A mobility, suggesting a release of molecules from the confinement of nanoclusters. Importantly, expression of syntaxinx1A^KARRA^-mEos2, a mutant with decreased affinity to PtdIns(3,4,5)*P*_3_, increased its mobility and prevented the activity-dependent diffusional changes elicited by thermogenetic stimulation. Furthermore, co-expression of tetanus toxin light chain (TeTx/LC) in *Drosophila* larval motor neurons increased Sx1A-mEos2 mobility, whereas a temperature-sensitive NSF mutation (comt^ST17^), which prevents SNARE complex disassembly^31^,^32^, had the opposite effect.

Taken together, our results suggest that the lateral diffusion and trapping of Sx1A in nanoclusters are altered by synaptic activity. We demonstrate that PtdIns(3,4,5)*P*_3_ mediates Sx1A trapping, and that SNARE complex disassembly favours free diffusion from nanoclusters.

## Results

### Imaging Sx1A-mEos2 mobility in motor nerve terminals *in vivo*

**Figure 1:**
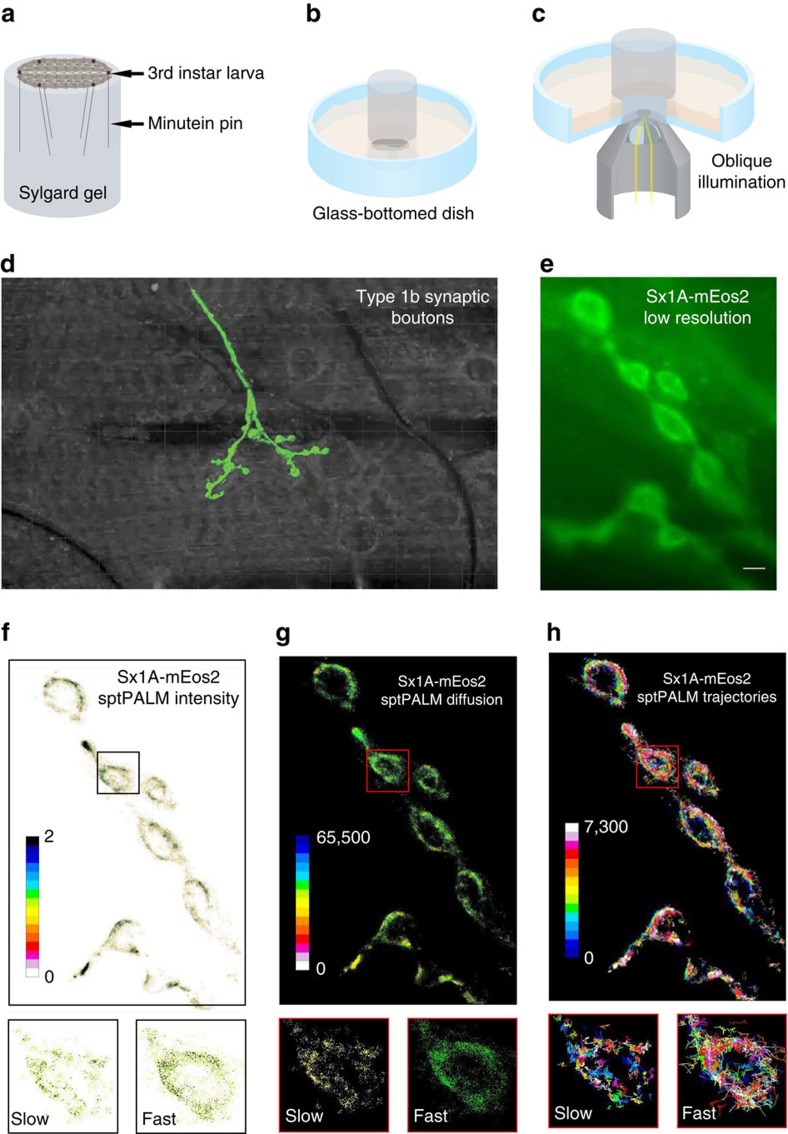
*In vivo* single-molecule imaging of Sx1A-mEos2 at fruit fly larval motor nerve terminals. Wide-field oblique illumination microscopy was used to carry out Sx1A-mEos2 single-molecule localization. Scheme representation of (**a**) filleted *Drosophila* third instar larva pinned onto sylgard gel using minutein pins, (**b**) the larva-sylgard preparation placed unto glass-bottomed dish filled with Schneider’s insect medium and (**c**) slightly oblique illumination used to visualize motor nerve terminals. (**d**) Type 1b synaptic boutons embedded in muscle 6 of the second abdominal segment of the fillet larvae were used for Sx1A-mEos2 imaging. (**e**) Low-resolution TIRF image of a live motor nerve terminal expressing Sx1A-mEos2 before photoconversion. Sx1A-mEos2 was imaged at 33 Hz for 7.5 min (25,000 frames) as indicated in the Methods section. (**f**–**h**) SptPALM average intensity, diffusion coefficient and trajectory map of Sx1A-mEos2 acquired from 6,127 individual trajectories. Scale bar, 5 μm. Inset: average intensity, diffusion coefficient and trajectory map showing slow and fast populations of Sx1A-mEos2 on a synaptic bouton.

To investigate whether Sx1A is organized into nanoclusters in live motor nerve terminals *in vivo*, we fused Sx1A with the photoconvertible fluorescent protein mEos2 (ref. 33) and then expressed the Sx1A-mEos2 construct in *Drosophila* using PhiC31-mediated integration^34^ (Supplementary Fig. 1a–c). These constructs carry the endogenous Sx1A promoter, thereby allowing expression in neurons, including motor neurons that are easily accessible in a filleted larval preparation (Fig. 1a). We therefore used Sx1A-mEos2 transgenic *Drosophila* larvae with full endogenous Sx1A expression. It should be noted that expression of Sx1A-mEos2 was quite low (Supplementary Fig. 1a–c) and not sufficient to rescue the null Sx1A mutant, as previously found with HA-Sx1A in the null background^24^. To visualize motor nerve terminals using total internal reflection fluorescence (TIRF) microscopy, we dissected third instar larvae that were held in place by minutien pins on a sylgard gel (see Methods). The gel was designed to fit in a glass-bottomed culture dish and the preparation was visualized with slightly oblique illumination so as to access the motor nerve terminals (Fig. 1a–c). To image the localization and mobility of Sx1A-mEos2, we used PALM (sptPALM)^35^. We could detect green-emitting fluorescence of the mEos2 tag clearly in motor nerve terminals (Fig. 1d,e). To resolve Sx1A single molecules in live motor nerve terminals, we used 405 nm laser beam illumination and imaged at 33 Hz in the red-emitting channel. Single molecules of Sx1A-mEos2 could be detected and analysed (Supplementary Movie 1) and a sptPALM intensity map was generated (Fig. 1f). Tracking individual molecules allowed us to generate maps showing the trajectories, as well as the instantaneous diffusion coefficients of Sx1A-mEos2 molecules (Fig. 1g,h). We could detect mobile and confined Sx1A (inset, Fig. 1f–h), defining nanoclusters reminiscent of those previously observed in neurosecretory cells^14^.

### Thermogenetic increase in synaptic activity raises Sx1A mobility

**Figure 2:**
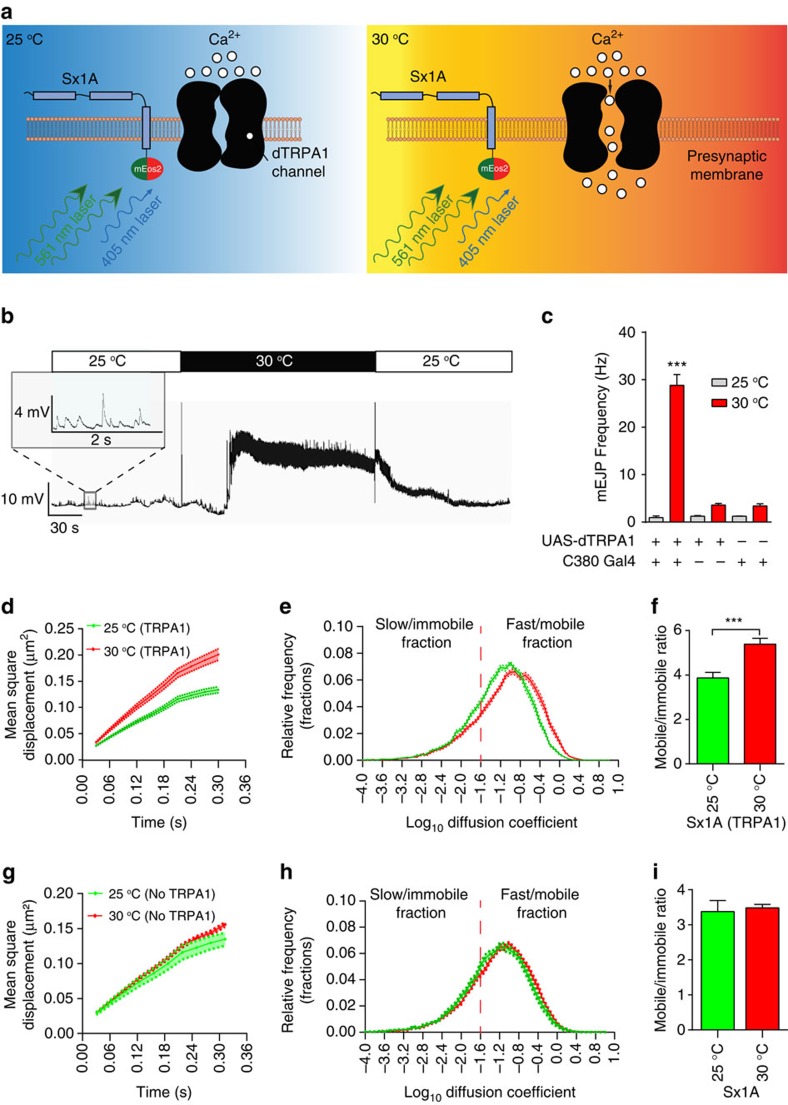
Thermogenetically induced presynaptic activity increases Sx1A mobility in live motor nerve terminals. Larvae expressing both Sx1A-mEos2 and dTRPA1 were imaged at 33 Hz in low (25 °C) and elevated temperatures (30 °C). (**a**) Schematic diagram of presynaptic membrane with Sx1A-mEos2 and dTRPA1 molecules. Sx1A-mEos2 was excited by the 561 nm laser and photoconverted by the 405 nm laser. The dTRPA1 channel is closed at 25 °C, but opens to allow calcium influx at 30 °C. (**b**) Electrophysiological recordings from abdominal wall muscle 6 of Sx1A-mEos2-expressing larvae with motor neuron expression of dTRPA1. Inset: miniature end plate potential recording at 25 °C. (**c**) Raising temperature from 25 to 30 °C significantly elevates the frequency of miniature end plate potentials (mEJP) (from 0.9±0.3 to 28.8±2.3 mEJP per s) in larvae expressing dTRPA1. Control UAS-dTRPA1 (25 °C, 1.2±0.1; and 30 °C, 3.6±0.3 mEJP per s) and control C380-Gal4 (25 °C, 1.2±0.1 mEJP per s; and 30 °C, 3.4±0.4 mEJP per s) larvae are comparatively unresponsive to increased temperature (*n*=3 different larvae for each). Comparisons were performed using one-way analysis of variance with Dunnett’s multiple comparison test ***P<0.001. (**d**–**f**) Change in MSD (μm^2^), relative frequency distribution of diffusion coefficients and ratio of mobile to immobile fractions of Sx1A-mEos2 elicited on thermogenetic stimulation. The mobile to immobile ratio increased from 3.9±0.3 to 5.4±0.3 (*n*=15 NMJ chains at 25 °C and 18 NMJ chains at 30 °C; average of ∼2,700 trajectories analysed per NMJ chain). The vertical dashed lines indicate the mobile to immobile threshold^38^. (**g**–**i**) Raising the temperature from 25 to 30 °C in larvae without dTRPA1 expression (yw, yellow white; Sx1A-mEos2; +/+) did not alter the MSD (μm^2^) and the distribution of the diffusion coefficients. The ratio of mobile to the immobile fractions was unchanged, from 3.4±0.3 at 25 °C to 3.5±0.1 at 30 °C (*n*=15 NMJ chains at 25 °C and 11 NMJ chains at 30 °C; average of ∼2,200 trajectories analysed per NMJ chain). Statistical tests were performed using the student's t test (two-tailed distribution, unpaired). ***, *P*<0.001. Mean±s.e.m. are plotted.

To investigate the effect of increasing synaptic transmission on Sx1A mobility at the neuromuscular junction (NMJ), we used a motor neuron-specific driver—C380-Gal4 (ref. 36) to express the heat-sensitive *Drosophila* transient receptor potential subfamily A1 (dTRPA1)^37^ on the motor nerve terminals of the larvae in which Sx1A-mEos2 was already constitutively expressed. These cation channels open when the ambient temperature rises from 25 to 30 °C (Fig. 2a)^37^, leading to an increase in spontaneous miniature excitatory junction potential (mEJP) frequency (Fig. 2b,c). This stimulatory effect was reversed by returning the temperature to 25 °C (Fig. 2b).

To quantify Sx1A mobility, we analysed the mean square displacement (MSD) of the sptPALM trajectories of individual Sx1A-mEos2 molecules to examine possible changes in mobility in response to the dTRPA1-induced increase in neurotransmitter release. Comparison of the MSDs between unstimulated and stimulated nerve terminals revealed a significant increase (Fig. 2d and Supplementary Fig. 2a). Analysis of the diffusion coefficient distribution of Sx1A-mEos2 molecules revealed the presence of two distinct populations: a slow/immobile fraction and a fast/mobile fraction. We calculated the threshold for Sx1A mobile and immobile states^27^ as previously described (see Methods)^38^. Sx1A-mEos2 mobility was significantly increased by stimulation (Fig. 2e and Supplementary Fig. 1d). This was most apparent when the mobile:immobile ratio was calculated (Fig. 2f). Importantly, this was not a temperature effect; control motor nerve terminals not expressing dTRPA1 did not exhibit any change in mobility or MSD and diffusion coefficient for the same temperature shift (Fig. 2g,h and Supplementary Figs 1e and 2b). In addition, the mobile:immobile ratio showed no significant change in the control (Fig. 2i).

### Diffusive states of Sx1A assessed by hidden Markov modelling

**Figure 3:**
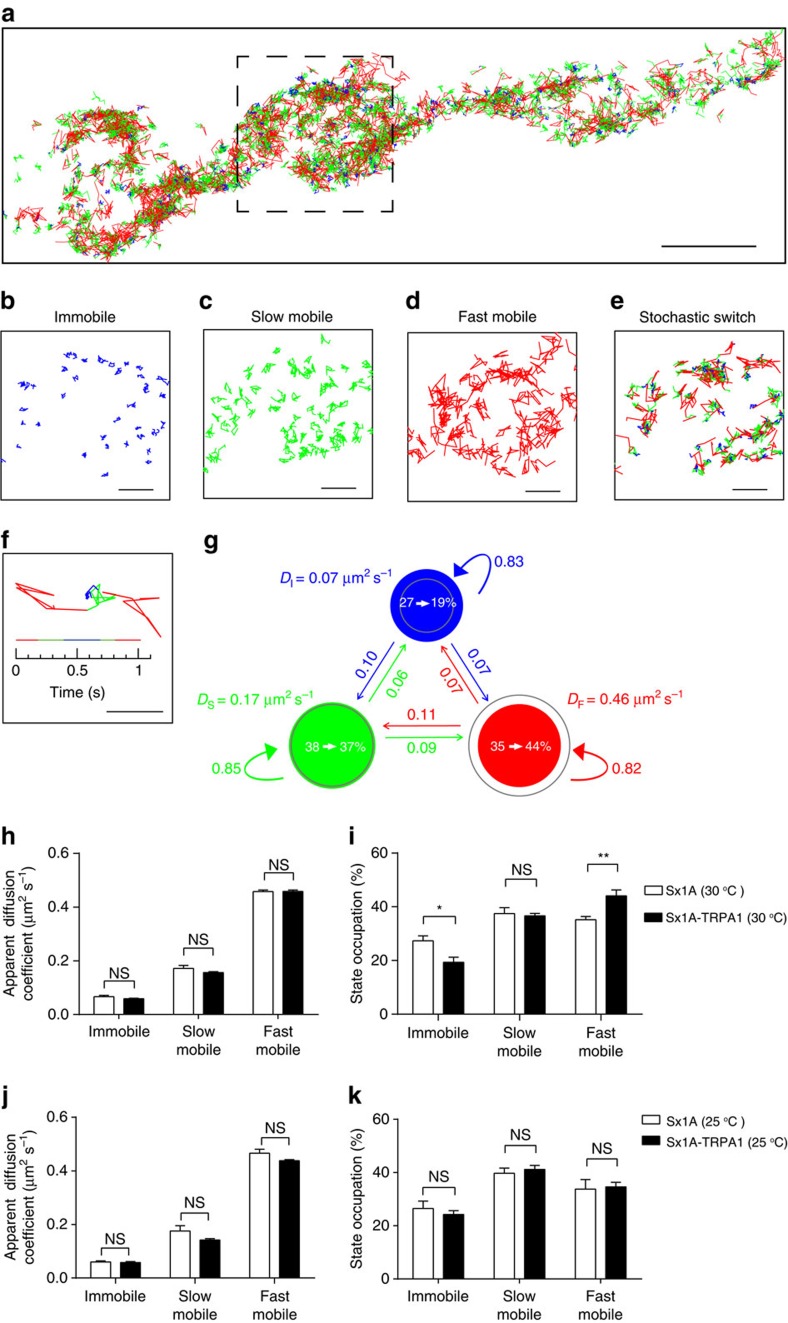
Hidden Markov modelling reveals the existence of distinct Sx1A-mEos2 diffusive states. (**a**) About 1,500 trajectories from one NMJ chain were colour-coded with the most likely diffusive state inferred by vbSPT analysis. Blue, green and red indicate immobile, slow mobile and fast mobile diffusive states, respectively. Scale bar, 3 μm. (**b**–**f**) A high-magnification view of colour-coded trajectories from the boxed region in **a**. Trajectories either continued to remain in the (**b**) immobile, (**c**) slow mobile or (**d**) fast mobile state, or (**e**) stochastically switched between different diffusive states. Scale bar, 1 μm. (**f**) An example trajectory undergoing stochastic switching between the three diffusive states. The timeline shows the inferred state occupation at different time points. Scale bar, 0.5 μm. (**g**) A three-state model and the parameters inferred by the vbSPT analysis of trajectories from NMJ chains at 30 °C without dTRPA1 expression. Circles represent the different states and are colour-coded as in **a**–**f**. The areas of the circle represent the average state occupation (%) of Sx1A-mEos2 in their respective states. Coloured arrows indicate the average transition probabilities between different diffusive states per time frame. *D*_I_, *D*_S_ and *D*_F_ are the average apparent diffusion coefficients of immobile, slow mobile and fast mobile states, respectively. Only changes in the state occupation (see white arrows within the circle) on dTRPA1 expression at 30 °C are shown. Grey circle areas correspond to state occupation of Sx1A-mEos2 in different states at 30 °C with dTRPA1 expression. (**h**–**j**) Apparent diffusion coefficients and state occupations inferred by analysing trajectories from NMJ chains with or without expression of dTRPA1 at (**h**,**i**) 30 and (**j**,**k**) 25 °C. Statistical tests were performed using Mann–Whitney *U*-test (NS, not significant, *P<0.05 and **P<0.01). *n*=12 and 11 NMJ chains at 25 and 30 °C without dTRPA1 expression, respectively, and 14 and 15 NMJ chains at 25 and 30 °C with dTRPA1 expression, respectively. Mean±s.e.m. are plotted.

We next used variational Bayes single-particle tracking (vbSPT) analysis to further investigate the effect of stimulation on Sx1A mobility^39^. The vbSPT analysis infers the number of hidden diffusive states and the state transition parameters from the experimental data based on Bayesian model selection applied to hidden Markov models, and annotates each step of the trajectories with the most likely diffusive states^39^. We found that there were three diffusive states (Fig. 3a–f; see Methods). On the basis of apparent diffusion coefficients of each state and MSD analysis of annotated trajectories (Supplementary Fig. 3), we categorized the three states as immobile (Fig. 3b), slow mobile (Fig. 3c) and fast mobile diffusive states (Fig. 3d). Importantly, we observed stochastic switching between the three diffusive states, highly suggestive of Sx1A moving in and out of Sx1A nanoclusters (Fig. 3e–g).

We next investigated how thermogenetic stimulation based on dTRPA1 expression affected the inferred parameters. Interestingly, raising synaptic activity with dTRPA1 expression at 30 °C only affected the state occupancy but not the apparent diffusion coefficients of each state (Fig. 3h,i). Indeed, the fast mobile state occupancy increased significantly at the expense of the immobile state occupancy, which significantly decreased. The slow mobile state occupancy did not change significantly in these conditions (Fig. 3i). We found that dTRPA1 expression at 25 °C did not significantly change the apparent diffusion coefficients (Fig. 3j) or the state occupancies (Fig. 3k). Furthermore, elevating temperature to 30 °C in the absence of dTRPA1 did not significantly affect these parameters (Supplementary Fig. 4). These results are consistent with our MSD and diffusion coefficient distribution analyses (Fig. 2). Overall, vbSPT analysis allowed us to reveal clear changes in state occupancy associated with the activity-dependent increase in Sx1A-mEos2 mobility. This demonstrates that Sx1A molecules constantly oscillate between these distinct diffusive states and that on stimulation, they spend less time in the immobile state and more in the mobile state.

### Optogenetic increase in synaptic activity raises Sx1A mobility

**Figure 4:**
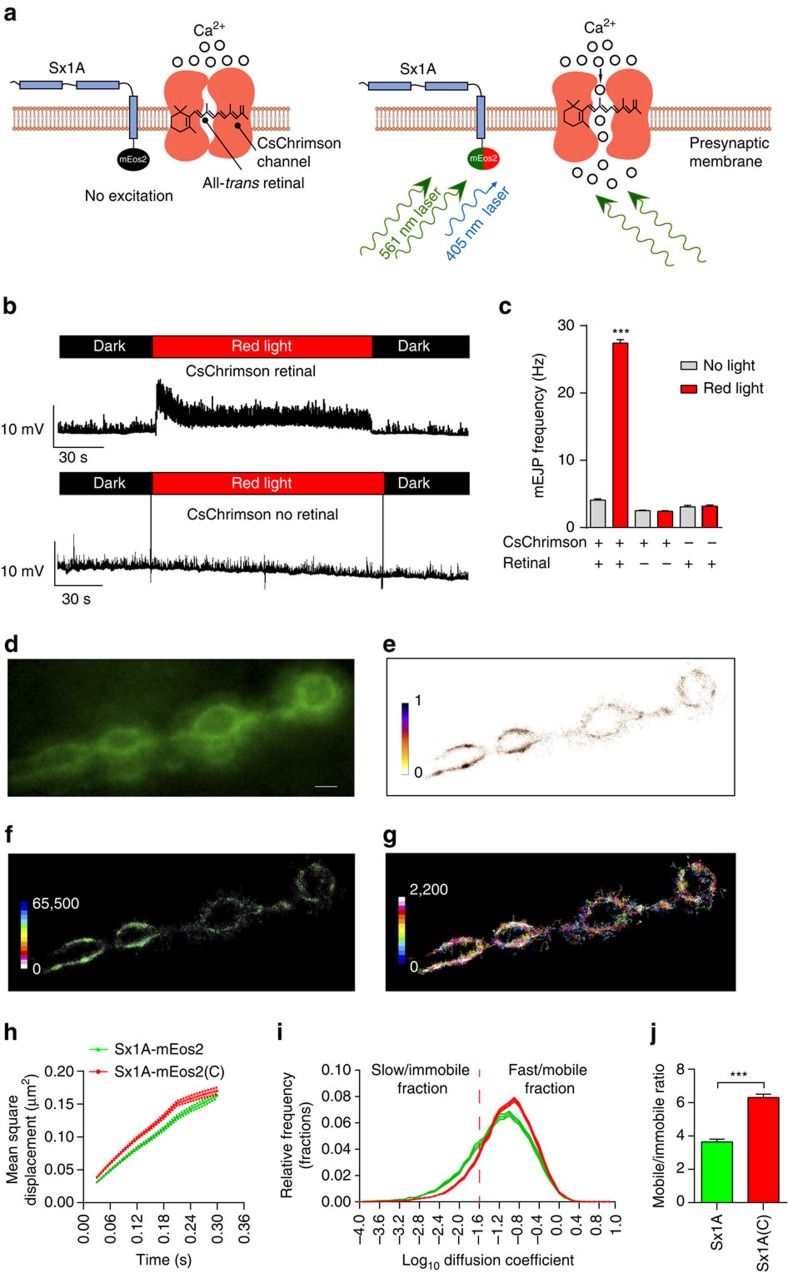
Optogenetic-induced rise in presynaptic activity increases Sx1A-mEos2 mobility in motor nerve terminals. Sx1A-mEos2 mobility was imaged at 33 Hz in larvae expressing the channelrhodopsin CsChrimson that had been fed with retinal. (**a**) Schematic diagram of presynaptic membrane with Sx1A-mEos2 and CsChrimson molecules. The excitation 561 nm beam used to image photoconverted Sx1A-mEos2 was simultaneously also used to elicit the opening of the CsChrimson cation channels. (**b**,**c**) Electrophysiological traces from the abdominal muscle 6 of CsChrimson- and Sx1A-mEos2-expressing larvae. Note that the mEJP frequency in retinal-fed larvae stimulated with far-red light is significantly higher (27.4±0.5 mEJPs per s) than that of control larvae with no light stimulation (4.1±0.2 mEJPs per s), no CsChrimson expression (far-red light, 3.1±0.2 mEJPs per s and no far-red light, 3.2±0.2 mEJPs per s) and not fed with retinal (far-red light, 2.42±0.10 mEJPs per s and no far-red light, 2.5±0.1 mEJPs per s). Comparisons were performed using one-way analysis of variance with Dunnett’s multiple comparison test ***P<0.001. (**d**–**g**) Representative low-resolution image, sptPALM average intensity, diffusion coefficient and trajectory map of Sx1A-mEos2 in CsChrimson-expressing larvae that had been fed with retinal (1,853 trajectories analysed). Scale bar, 5 μm. (**h**,**i**) Note that optogenetic-induced presynaptic activity increased the MSD and the diffusion coefficient distribution. C, CsChrimson. (**j**) The ratio of mobile to immobile fractions significantly increased from 3.6±0.15 to 6.31±0.2 (*n*=21 NMJ chains for Sx1A-mEos2 and CsChrimson-retinal and 19 NMJ chains for Sx1A-mEos2 only; average of ∼3,200 trajectories analysed per NMJ chain). Statistical tests were performed using the student's *t* test (two-tailed distribution, unpaired). ***, *P*<0.001. Mean±s.e.m. are plotted.

We next turned to an optogenetic stimulation approach to ensure that the activity-dependent increase in Sx1A mobility was independent of the way in which the motor nerve terminals were stimulated. We expressed CsChrimson, a far-red shifted homologue of channelrhodopsin^40^, at the motor nerve terminal using the motor neuron-specific Gal4 driver C380-Gal4 in a Sx1A-mEos2 background. In the presence of retinal and red light (see Methods), the transgenic channel opens, thereby allowing cation influx (Fig. 4a) and triggering neurotransmitter release^40^. We confirmed that CsChrimson activation led to increased neurotransmitter release by electrophysiology, and detected a marked increase in the frequency of spontaneous mEJPs on far-red light exposure (Fig. 4b). Such an increase in mEJP frequency was solely detected in retinal-fed larvae that were illuminated (Fig. 4c). As the 561 nm photoactivation laser beam needed to activate mEos2 falls within the activation spectral range for the CsChrimson channel^40^, we were therefore able to assess the effects of activation on Sx1A-mEos2 mobility simultaneously. Consistent with our previous result, Sx1A-mEos2 mobility increased upon optogenetic-induced synaptic transmission (Fig. 4d–j and Supplementary Fig. 2c). Analysis of the diffusion coefficient distribution also confirmed an overall change upon optogenetic stimulation and revealed a decreased slow/immobile fraction accompanied by an increase in the mobile fraction (Fig. 4i and Supplementary Fig. 1f), consistent with our dTRPA1 results. The mobile:immobile ratio was also significantly increased in response to light-induced CsChrimson-mediated synaptic activity (Fig. 4j).

### Sx1A is organized in nanoclusters *in vivo*

**Figure 5:**
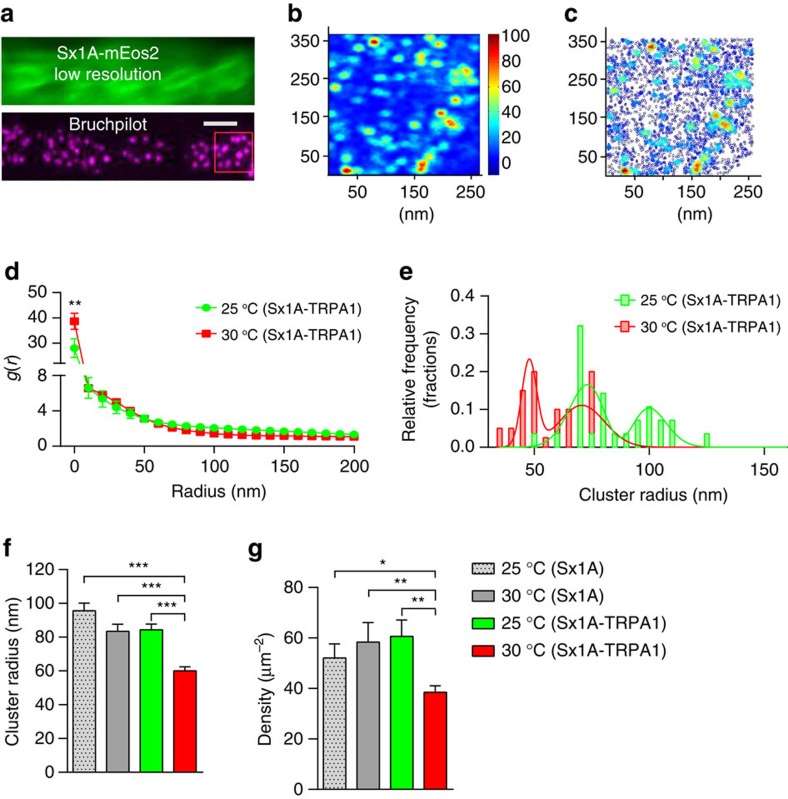
Thermogenetic-induced rise in presynaptic activity leads to a decrease in Sx1A-mEos2 cluster size and density. Live larvae expressing Sx1A-mEos2 and dTRPA1 were imaged at low resolution to locate NMJs, after which the preparation was paraformaldehyde fixed, rinsed and processed for bruchpilot immunocytochemistry before single-molecule localization of Sx1A-mEos2 on the same NMJ. (**a**) Low-resolution image of a Sx1A-mEos2-expressing live motor nerve terminal and bruchpilot active zone immunostaining of the same nerve terminal. Scale bar, 5 μm. (**b**,**c**) Cluster map colour-coded for cluster size and molecule density distribution of Sx1A-mEos2 generated from Ripley’s K-function. (**d**) Average of auto-correlation functions of each condition was fitted using equation (3). In all, 39 regions of interest (ROIs) were used for unstimulated larvae and 29 ROIs for stimulated larvae. A minimum of 3 different larvae was used in each condition. (**e**) Cluster radius and density were obtained from fitting the auto-correlation values. In unstimulated Sx1A-mEos2 larvae expressing dTRPA1, two peak cluster sizes were observed at 73.2±1.8 and 100±1.89 nm at 25 °C. These decreased to 47.8±0.5 and 70.7±1.6 nm when the temperature was increased to 30 °C. (**f**,**g**) The average cluster radius was 83.5±4.2 nm in unstimulated Sx1A-mEos2 motor nerve terminals expressing dTRPA1; this significantly decreased to 60.1±2.4 nm in stimulated terminals. In larvae expressing only Sx1A-mEos2, at 25 °C, the average cluster radius were 95.6±4.5 and 84.5±3.37nm at 30 °C. The average density of Sx1A-mEos2 significantly decreased from 60.9±6.5 to 38.5±2.6 μm^−2^ with elevated synaptic activity. Statistical tests were performed using the Student’s *t*-test (two-tailed distribution, unpaired). *P<0.05, **P<0.01, ***P<0.001. Mean±s.e.m. are plotted.

Sx1A is distributed in nanoclusters *in vitro*^13^. We therefore questioned whether the increase in Sx1A-mEos2 mobility associated with elevated levels of synaptic transmission could lead to a decrease in both the number of molecules per nanocluster and in the average size of the nanoclusters. We addressed this question by implementing a single-molecule localization experiment and cluster analysis in fixed motor nerve terminals co-expressing Sx1A-mEos2 and dTRPA1. To carry out stimulation, larvae expressing dTRPA1 were incubated at 30 °C for 5 min before fixation. Control-unstimulated experiments were carried out on larvae devoid of dTRPA1 expression at 25 and 30 °C. To accurately sample localization within nerve terminals, an active zone marker (bruchpilot) was used (Fig. 5a). Sx1A-mEos2 labelling was largely punctate (Fig. 5b), in good agreement with a nanocluster organization^13^,^24^. Cluster maps, created by calculating Ripley’s K function^41^,^42^, also confirmed the cluster organization of Sx1A-mEos2 molecules (Fig. 5c). We used a pair-correlation analysis^43^,^44^ to quantify the tendency of Sx1A-mEos2 to cluster (Fig. 5d and Supplementary Fig. 5a). Clustering was determined by fitting auto-correlation values to a function that accounted for over-counting of localizations with finite resolution. Before stimulation (25 °C), the population distribution for Sx1A-mEos2 cluster radius peaked at 73.2±1.9 and 100±1.9 nm (mean±s.e.m.). However, the radius of both populations decreased on elevated presynaptic activity (30 °C) with two peak populations being observed at 47.8±0.5 and 70.7±1.6 nm (mean±s.e.m.), respectively (Fig. 5e and Supplementary Fig. 5b). At rest (25 °C), Sx1A-mEos2 clusters had an average radius of 95.6±4.5 nm (mean±s.e.m.). Similar values were obtained with the other rest controls: (30 °C no dTRPA1) 84.5±3.4 nm and (25 °C with dTRPA1) 83.4±4.2 nm (Fig. 5f) (mean±s.e.m.). On stimulation, the average cluster radius significantly decreased to 60.1±2.4 nm (mean±s.e.m.), thereby confirming our hypothesis that Sx1A molecules are released from nanoclusters as a result of stimulation. Consistent with this idea, the number of Sx1A-mEos2 molecules per cluster significantly decreased to 31.5±3.1 (mean±s.e.m.) on stimulation (Supplementary Fig. 5c). Furthermore average Sx1A density decreased after stimulation from 60.9±6.5 to 38.5±2.6 μm^−2^ (Fig. 5g) (mean±s.e.m.). Importantly, the number of molecules per cluster and the cluster density were unaffected by raising the temperature in controls lacking TRPA1 channels (Fig. 5g and Supplementary Fig. 5c).

### Polyphosphoinositide controls activity-dependent release of Sx1A

**Figure 6:**
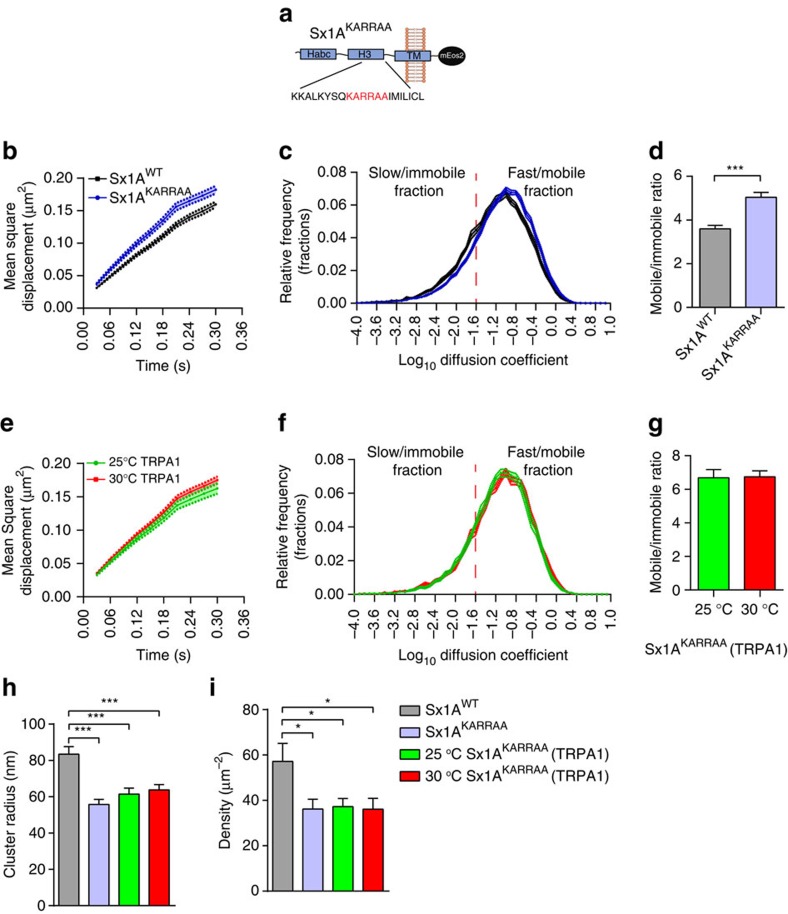
PtdIns(3,4,5)*P*_3_ interaction alters Sx1A mobility. (**a**) Scheme illustrating the Sx1A^KARRAA^ mutation that is known to exhibit reduced affinity to PtdIns(3,4,5)*P*_3_. The Sx1A^KARRAA^ mutation was transgenically expressed in *Drosophila* and sptPALM was carried out on live motor nerve terminals. (**b**,**c**) Comparison of MSDs and diffusion coefficient distributions of Sx1A-mEos2 and Sx1A^KARRAA^-mEos2 (*n*=17 NMJ chains for Sx1A-mEos2 and 19 NMJ chains for Sx1A^KARRAA^-mEos2; average of ∼2,600 trajectories analysed per NMJ chain). (**d**) The mobile to immobile ratio was 3.6±0.2 for Sx1A-mEos2 and 5.0±0.2 for Sx1A^KARRAA^-mEos2. (**e**,**f**) Sx1A^KARRAA^-mEos2 or Sx1A-mEos2 larvae with concomitant dTRPA1 expression were heated from 25 to 30 °C to stimulate neurotransmitter release. Note that the MSD of Sx1A^KARRAA^-mEos2 was also not affected by stimulation. (**g**) The mobile to immobile ratio was not significantly altered: 6.7±0.5 at 25 °C to 6.7±0.3 at 30 °C (*n*=13 NMJ chains at 25 °C and 14 NMJ chains at 30 °C; average of ∼1,700 trajectories per NMJ chain). (**h**) Fixed larvae were analysed for SML. The average cluster radius of Sx1A^KARRAA^-mEos2 (55.8±2.7 nm) compared with Sx1A-mEos2 (83.5±4.2 nm) was significantly different. Expressing dTRPA1 (25 °C, 61.5±3.3 nm) or elevating the temperature did not significantly alter the cluster size (30 °C, 63.7±2.9 nm). (**i**) The Sx1A^KARRAA^-mEos2 average density was significantly lower (36.1±4.3 μm^−2^) than that of Sx1A-mEos2 (57.2±7.9 μm^−2^); this was unchanged by dTRPA1 expression (25 °C, 37.3±3.6/μm^2^) and stimulation (30 °C, 36.1±4.8 μm^−2^). Statistical tests were performed using Student’s *t*-test (two-tailed distribution, unpaired). *P<0.05, ***P<0.001. Mean±s.e.m. are plotted.

Sx1A clustering has been shown to be promoted by phosphoinositides^22^,^23^,^45^,^46^. Notably, PtdIns(3,4,5)*P*_3_ interaction with Sx1A juxta-membrane lysine residues has been reported to control Sx1A localization to active zones, and mutating these residues to alanine (KARRAA mutation) drastically decreases synaptic transmission^24^. We therefore hypothesized that this mutation should interfere with the activity-dependent change in Sx1A mobility. To address this, we carried out sptPALM on *Drosophila* larvae expressing Sx1A^KARRAA^-mEos2 (Fig. 6a and Supplementary Fig. 6a). As expected, this mutation conferred a higher mobility to Sx1A molecules compared with the wild-type Sx1A-mEos2 (Fig. 6b and Supplementary Fig. 2d). Analysis of the diffusion coefficient distribution and the mobile:immobile ratio revealed that Sx1A^KARRAA^-mEos2 was significantly more mobile than wild-type Sx1A-mEos2 (Fig. 6c,d). We then questioned whether this mutation could also affect the activity-dependent change in Sx1A mobility detected on raising synaptic activity (Fig. 2). We assayed for this by stimulating the motor neuron of Sx1A^KARRAA^-mEos2 larvae thermogenetically with dTRPA1 expression. As expected, the mobility of Sx1A^KARRAA^-mEos2 was unaltered by stimulation (Fig. 6e and Supplementary Fig. 2e). Analysis of the diffusion coefficient distribution as well as the mobile:immobile ratio revealed no change in mobility of Sx1A^KARRAA^-mEos2 (Fig. 6f,g). In addition, we observed that the mobility of Sx1A^KARRAA^-mEos2 in the absence of dTRPA1 was unaltered by increasing the temperature from 25 to 30 °C (Supplementary Fig. 6b–d).

We then measured the cluster size and number of Sx1A^KARRAA^-mEos2 molecules per cluster as described above (Fig. 6h and Supplementary Fig. 6e,f). Compared with Sx1A-mEos2, the Sx1A^KARRAA^-mEos2 cluster radius was significantly lower at 55.8±2.7 nm (mean±s.e.m.) and this was unchanged by thermogenetic stimulation (Fig. 6h). Similarly, the average Sx1A^KARRAA^-mEos2 density and the number of molecules per cluster were significantly lower compared with Sx1A-mEos2 and these were unchanged by stimulation (Fig. 6i and Supplementary Fig. 6e,f). These data demonstrate that the interaction with PtdIns(3,4,5)*P*_3_ controls the trapping of Sx1A molecules in nanoclusters. Indeed, the number of molecules per cluster, their density and their activity-dependent release from these confinement sites are affected by PtdIns(3,4,5)*P*_3_.

### Sx1A mobility increases on TeTx/LC expression

**Figure 7:**
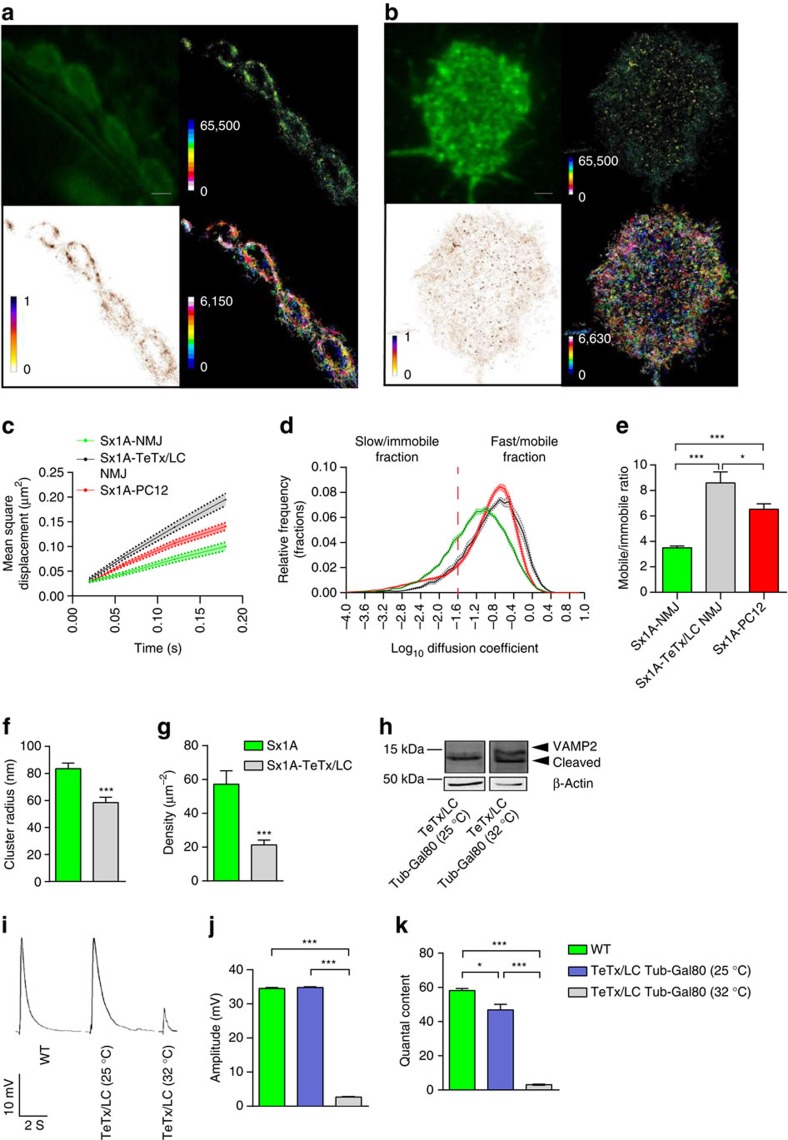
Sx1A-mEos2 mobility is increased by co-expression of TeTx/LC in motor nerve terminals. TeTx/LC was transiently expressed in Sx1A-mEos2-expressing larvae. Single-molecule tracking was then carried out on the motor nerve terminals. (**a**) Representative low-resolution image, sptPALM average intensity, diffusion coefficient and trajectory map of Sx1A-mEos2 in larvae with motor neuron expression of TeTx/LC (1,836 trajectories analysed). Scale bar, 3 μm. (**b**) Low-resolution image, sptPALM average intensity, diffusion coefficient and trajectory map of Sx1A-mEos2 in PC12 cells. (**c**–**e**) Blocking SNARE complex formation via TeTx/LC expression increased the MSD, the diffusion coefficient distribution and the ratio of the mobile to immobile fractions of Sx1A-mEos2 in live motor nerve terminals (Sx1A-mEos2 at the NMJ, 3.5±0.1; Sx1A-mEos2 with TeTx/LC expression at the NMJ, 8.6±0.8; and Sx1A-mEos2 in PC12 cells, 6.5±0.4). This increase in Sx1A-mEos2 mobility is comparable to that observed in PC12 cells (*n*=19 NMJ chains for Sx1A-mEos2-expressing larvae, *n*=14 NMJ chains for larvae also expressing TeTx/LC and *n*=23 cells for PC12; average of ∼3,000 trajectories in NMJ chains and ∼11,500 trajectories in PC12 cells). Comparisons were performed using one-way analysis of variance (ANOVA) with Tukey’s multiple comparison test. *P<0.05, ***P<0.001. (**f**,**g**) TeTx/LC expression significantly decreased the Sx1A-mEos2 cluster size from 83.5±4.2 to 58.4±4.0 nm. The average Sx1A-mEos2 density also decreased significantly from 57.2±7.9 to 21.2±2.9 μm^−2^. Statistical tests were performed using the Mann–Whitney *U*-test ***P<0.001. (**h**) Western blot showed that VAMP2 was cleaved by TeTx/LC expression at 32 °C; at 25 °C, there was no TeTx/LC expression and VAMP2 remained intact. (**i**–**k**) Excitatory junction potentials in wild-type *Drosophila* larvae, Sx1A-mEos2 transgenic larvae with tubulin Gal80 suppression of TeTx/LC (25 °C) and Sx1A-mEos2 larvae with TeTx/LC expression (32 °C); the amplitude of evoked release decreased significantly on TeTx/LC expression (wild type, 35.3±0.2 mV; TeTx/LC suppression at 25 °C, 35.2±0.1 mV; and TeTx/LC expression at 32 °C, 2.6±0.2 mV). The quantal content decreased significantly with the expression of TeTx/LC (wild type, 58.1±1.0; TeTx/LC suppression at 25 °C, 46.8±3.2; and TeTx/LC expression at 32 °C, 3.1±0.3). Comparisons were performed using one-way ANOVA with Tukey’s multiple comparison test *P<0.05, ***P<0.001. Mean±s.e.m. are plotted. WT, wild type.

We speculated that because motor nerve terminals exhibit a large population of docked and primed synaptic vesicles^47^, Sx1A-mEos2 mobility might be affected by preassembled *trans*-SNARE complexes. Therefore, the increase in Sx1A-mEos2 mobility detected on stimulation could result from the release of Sx1A-mEos2 molecules previously engaged in *trans*-SNARE complexes^15^,^27^. To test this hypothesis, we transiently expressed TeTx/LC, which cleaves VAMP2 and prevents SNARE complex formation^48^. Expression of TeTx/LC in the fruit fly is embryonic lethal^3^, we therefore suppressed the expression of TeTx/LC from the embryo stage to the third instar larva stage using a temperature-sensitive tubulin Gal80 (ref. 49). TeTx/LC was expressed by incubating the larvae at 32 °C for 4 h before carrying out sptPALM to assess Sx1A-mEos2 mobility. Sx1A-mEos2 mobility in PC12 cells was also assessed for comparative purposes (Fig. 7a,b). The MSD of Sx1A-mEos2 molecules significantly increased in motor nerve terminals following expression of TeTx/LC (Fig. 7c). Analysis of the diffusion coefficient distribution and the mobile:immobile ratio showed an increase in the mobility of Sx1A-mEos2 as a result of TeTx/LC expression (Fig. 7d,e). Interestingly, the distribution of Sx1A-mEos2 diffusion coefficients was similar to that observed in neurosecretory cells (PC12 cells), which are known to have many fewer primed vesicles compared with live motor nerve terminals^50^. In addition, we observed that at 25 °C, when TeTx/LC is suppressed, Sx1A-mEos2 mobility was unaltered, as shown by diffusion coefficient analysis and the mobile:immobile ratio (Supplementary Fig. 7a,b).

Furthermore, we measured the cluster size and density of Sx1A-mEos2 on VAMP2 cleavage by TeTx/LC (Fig. 7f,g). The cluster size was reduced significantly to 58.4±4.0 nm (Fig. 7f) (mean±s.e.m.) and the average number of Sx1A-mEos2 molecules per cluster was significantly reduced to 15.8±4.2 (Supplementary Fig. 7c) (mean±s.e.m.). In addition, two cluster size peaks were observed at 52±2.0 and 88.5±0.9 nm (Supplementary Fig. 7d) (mean±s.e.m.). The average density of Sx1A-mEos2 was significantly reduced to 21.3±3.0 μm^−2^ (Fig. 7g) (mean±s.e.m.). We confirmed by western blotting that heat suppression of tubulin Gal80 elicited VAMP2 cleavage on TeTx/LC expression (Fig. 7h) and blockade of neurotransmitter release (Fig. 7i–k and Supplementary Fig. 7e).

### Sx1A mobility is reduced by expression of NSF comt^ST17^

**Figure 8:**
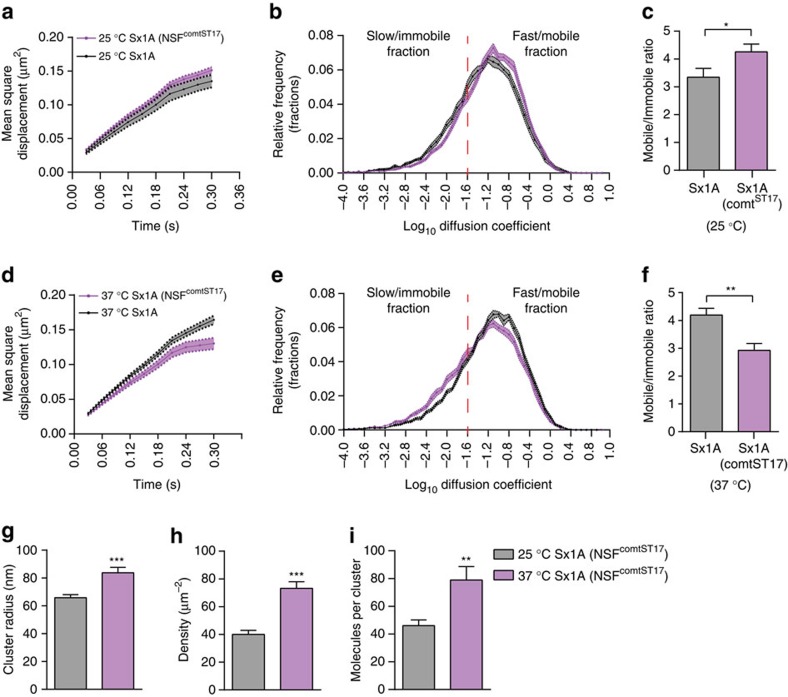
NSF comt^st17^ heat-sensitive inactivation decreases Sx1A-mEos2 mobility. Sx1A-mEos2 transgenic lines were expressed in the background of the comatose mutant of NSF (comt^ST17^). SptPALM was carried out on male larvae at a lower temperature (25 °C) when comt^ST17^ is functional, and at a higher temperature (37 °C) when it is inactivated. (**a**–**c**) At 25 °C, the MSD, diffusion coefficient distribution and the mobile to immobile ratio of the comt^ST17^-expressing Sx1A-mEos2 was slightly increased (*n*=15 NMJ chains for Sx1A-mEos2 at 25 °C and 13 NMJ chains when NSF comt^ST17^ was functional at 25 °C; average of ∼1,900 trajectories were analysed per NMJ chain). (**d**,**e**) Note that NSF comt^ST17^inactivation at 37 °C reduced Sx1A-mEos2 mobility (MSD and diffusion coefficient distribution). (**f**) The mobile to immobile ratio was significantly decreased from 4.2±0.2 to 2.9±0.2 on inactivation of NSF comt^ST17^ (*n*=17 NMJ chains for Sx1A-mEos2 at 37 °C and 12 NMJ chains for Sx1A-mEos2 when NSF comt^ST17^ was inactivated at 37 °C; average of ∼2,200 trajectories analysed per NMJ chain). (**g**–**i**) Inactivation of NSF comt^ST17^ significantly increased Sx1A-mEos2 cluster size from 65.8±2.2 to 83.7±3.9 nm. The average Sx1A-mEos2 density also increased significantly from 39.97±2.9 to 73.2±4.7 μm^−2^. The average number of Sx1A-mEos2 molecules per cluster significantly increased from 46.1±4.1 to 78.9±9.6 on NSF comt^ST17^ inactivation (*n*=35 NMJ chains for each condition). Statistical tests were performed using the Student’s *t* test (two-tailed distribution, unpaired). *P<0.05, **P<0.01, ***P<0.001. Mean±s.e.m. are plotted.

NSF and α-SNAP are responsible for promoting SNARE complex disassembly^51^. We therefore investigated whether the activity-dependent increase in Sx1A-mEos2 mobility stems from disengagement following disassembly of the SNARE complex using the temperature-sensitive NSF comt^ST17^ (comatose) mutant. This mutation prevents the disassembly of the SNARE complex^31^,^32^,^52^ in a temperature-controlled manner. When functional at room temperature (25 °C), Sx1A-mEos2 mobility increased slightly (Fig. 8a–c and Supplementary Fig. 2f). However, when the temperature was increased to 37 °C thereby inactivating NSF comt^ST17^, Sx1A-mEos2 was inactivated, Sx1A-mEos2 mobility significantly decreased (Fig. 8d and Supplementary Fig. 2g). Analysis of the diffusion coefficient also showed a significant increase in the immobile fraction of Sx1A-mEos2 with a corresponding decrease in the mobile fraction (Fig. 8e). Consequently, the mobile:immobile ratio showed a significant decrease on NSF comt^ST17^ inactivation (Fig. 8f). Accordingly, measurement of Sx1A-mEos2 cluster size, density and molecules per cluster on NSF comt^ST17^ inactivation revealed a significant increase in these measures (Fig. 8g–i) These data strongly suggest that SNARE disassembly elicited by stimulation is responsible for the Sx1A-mEos2 activity-dependent increase in mobility. Sx1A nanoclusters are therefore likely to contain pre-assembled SNARE complexes that are released from their trap on NSF-mediated SNARE complex disassembly.

Our results demonstrate that PtdIns(3,4,5)*P*_3_ and synaptic activity exert opposing effects on the lateral trapping of Sx1A in nanoclusters. Furthermore, Sx1A immobility at rest is mainly dependent on pre-engagement in the SNARE complex, and the activity-dependent increase in mobility relies on NSF-dependent disassembly of the SNARE complex after synaptic vesicle fusion.

## Discussion

In this study, we have used single-molecule imaging analysis in live presynaptic terminals to investigate the lateral mobility of Sx1A. To the best of our knowledge our study is the first investigation into the mobility of a SNARE protein *in vivo.* By expressing mEos2-tagged Sx1A in *Drosophila* using a Sx1A endogenous promoter, we have been able to access the mobility of individual molecules by sptPALM. We found that the dynamic interplay between the mobile and immobile fractions of Sx1A was significantly affected by increasing neurotransmission. Using light-activated CsChrimson—a far-red shifted channelrhodopsin^40^—or heat-activated dTRPA1 (ref. 37) to increase synaptic activity, we detected an overall increase in Sx1A mobility. Using vbSPT^39^, we could demonstrate that this increase stemmed from a double switch in occupancy states characterized by a reduced occupancy in the immobile state and an increased occupancy in the mobile state. Using single-molecule localization and cluster analysis from fixed preparations, we found that increasing synaptic activity was associated with a reduction in the number of Sx1A molecules per nanocluster, as well as a reduction in nanocluster size and density. Importantly, we report that interfering with Sx1A PtdIns(3,4,5)*P*_3_ binding increased Sx1A mobility, and prevented the activity-dependent change in Sx1A mobility; the number of molecules per nanocluster, size of nanoclusters and density were also reduced. In addition, we demonstrate that co-expression of TeTx/LC increases Sx1A mobility strongly, suggesting that at least some of the Sx1A nanoclusters contained pre-assembled SNARE complexes from primed synaptic vesicles as previously suggested^14^,^15^,^29^. Finally, Sx1A-mEos2 mobility was reduced when expressed in the background of an NSF mutant that prevents the disassembly of the SNARE complex. Our results suggest that some of the Sx1A molecules within these clusters are pre-engaged in *trans*-SNARE complex formation and that stimulation elicits their lateral diffusion, presumably from the *cis*-SNARE complex following the action of NSF and α-SNAP.

Our study reveals that increasing neurotransmitter release significantly impacts the lateral diffusion and reversible trapping of one of the SNAREs in a living organism. Previous works on membrane sheets and cultured neurosecretory cells have established that Sx1A is organized in nanoclusters^13^,^16^,^29^. Sx1A clustering was shown to mainly rely on weak homotypic interactions with the SNARE motif, as well as binding to specific phosphoinositides^22^,^23^,^24^. The average Sx1A cluster size found in the motor nerve terminals of *Drosophila* larvae was larger than previously reported in membrane sheets and neurosecretory cells^14^,^29^. Interestingly, the size distribution indicated two distinct populations, which were differentially affected by increasing synaptic activity. We detected an increase in the proportion of smaller clusters, similar to that previously reported^13^. As docking of secretory vesicles has previously been shown to be capable of recruiting Sx1A molecules^29^, it is possible that larger clusters represent sites of docked and primed synaptic vesicles. It is tempting to speculate that following extensive exocytic fusion events, a proportion of Sx1A molecules will be released from these nanoclusters, thereby reducing their size. In addition, the presence of larger clusters formed from aggregation of smaller clusters has previously been detected in neurosecretory cells^16^. It is worth noting that the expression level of the Sx1A-mEos2 construct was low and unable to rescue the homozygous Sx1A null mutant, raising the possibility that its expression could have compromised the final fusion step of exocytosis. It is also possible that mEos2 is prone to aggregation^53^, and more work using the newer mEos3.1 and 3.2 should be carried out to clarify whether mEos2 influences the clustering of Sx1A. However, despite these caveats, our data clearly demonstrate that Sx1A-mEos2 can form clusters, which are affected by stimulation and various treatments. It is therefore likely that, at the expression level used, Sx1A-mEos2 provides a valuable tool for detecting Sx1A clustering and mobility changes.

We also found that other factors influenced the lateral trapping of Sx1A in nanoclusters. A stretch of lysine residues at the end of the Sx1A H3-domain have been shown to control nanocluster formation via an interaction with PtdIns(4,5)*P*_2_ (ref. 46) and PtdIns(3,4,5)*P*_3_ (ref. 24). Indeed mutating these residues strongly reduced the ability of Sx1A to form nanoclusters in *Drosophila* motor nerve terminals^24^ and in PC12 membrane sheets^46^. Furthermore, this mutation was shown to mis-localize Sx1A from the active zones of motor nerve terminals of *Drosophila* and to decrease synaptic transmission^24^. Our data using Sx1A^KARRAA^ confirm the critical role of these residues in Sx1A nanocluster formation. More importantly, we demonstrate that mutating these residues prevents the lateral trapping of Sx1A in nanoclusters at rest and the ability of nanoclusters to release Sx1A molecules by lateral diffusion on stimulation. This demonstrates that the interaction with PtdIns(3,4,5)*P*_3_ plays a key role in the engagement of Sx1A into the SNARE complex during synaptic transmission. Our data suggests that interfering with this interaction reduces the availability of Sx1A to form the SNARE complex. Future work will be needed to assess the selectivity of Sx1A binding to PtdIns(4,5)*P*_2_ (ref. 46) and PtdIns(3,4,5)*P*_3_ (ref. 24), and whether stimulation affects these binding properties. A recent study from our group demonstrated that converting PtdIns(3,4,5)*P*_3_ to PtdIns(4,5)*P*_2_ is essential to promote secretory vesicle docking and priming^54^. An activity-dependent change in the relative binding of Sx1A to these two lipids within the confinement of Sx1A nanoclusters may therefore also contribute to release of Sx1A by lateral diffusion.

Our data show that the interplay between the mobile and immobile fractions of Sx1A is dynamically controlled by SNARE-dependent exocytosis. Interestingly, previous work performed using fluorescence recovery after photobleaching of cultured hippocampal neurons, reported no change in Sx1A mobility associated with the use of calcium chelators to lower activity^27^. However, a more recent study did find an increase in Sx1A mobility elicited by calcium buffering, although the mechanism underpinning this change was not investigated^28^. Our results using both thermogenetic and optogenetic stimulation showed a significant increase in Sx1A mobility that was sensitive to VAMP2 cleavage elicited by co-expression of TeTx/LC. Because motor nerve terminals contain a significant number of docked and primed synaptic vesicles^55^, we posit that the activity-dependent increase in Sx1A mobility could result from the SNARE-dependent fusion of these vesicles and the release of free Sx1A following NSF and α-SNAP unzipping of the *cis*-SNARE complex. Our results showing that the NSF-deficient comatose mutant^31^,^32^ significantly reduced Sx1A mobility strongly supports this view. Changes in the size and density of Sx1A clusters due to NSF comt^ST17^ inactivation further suggest that Sx1A clustering is also strongly affected by the activity of NSF and α-SNAP. Indeed, previous studies have revealed an accumulation of *cis*-SNARE complexes in *D. melanogaster* comt mutants exposed to restrictive temperatures^32^,^56^. Other factors may also control Sx1A mobility and trapping in nanoclusters, such as Munc18-1 (refs 57, 58) and other known Sx1A binders^59^,^60^,^61^. More work will be needed to further characterize the role of these factors in trapping Sx1A.

Our results suggest that the relatively low mobility of Sx1A molecules found in resting nerve terminals is caused by the fact that a significant number of Sx1A molecules are pre-engaged in SNARE complexes. The degree of Sx1A immobility in resting nerve terminals is therefore likely to be indicative of the number of primed synaptic vesicles. Phosphoinositide binding also largely contributes to the lateral trapping of Sx1A molecules in nanoclusters. During stimulation, Sx1A molecules pre-engaged in the SNARE complex are freed and undergo lateral diffusion in an NSF-dependent manner, suggesting that nanoclusters are areas of the plasma membrane that are capable of dynamically controlling the trapping and release of Sx1A molecules in an activity-dependent manner.

## Methods

### Molecular biology

Sx1A-mEos2 and Sx1A^KARRAA^-mEos2 were constructed by *in vivo* recombination in *Saccharomyces cerevisiae* using the pFL44S(W+)-attB-HA-Sx1A^**WT**^ vector^24^,^62^. This vector was linearized with SpeI and co-transformed into yeast cells together with a partially overlapping PCR fragment amplified from the pmEos2-N1 plasmid designed so as to fuse the mEos2 tag to the Sx1A C terminus. Primers used are listed in Supplementary Table 1. Recombined constructs were sequenced and stable transgenic fly lines were generated using PhiC31-mediated integration^34^ on the second chromosome (2L: 5108448, attP40) by injecting the constructs into *Drosophila* embryos (Best Gene). The endogenous Sx1A promoter was used to drive the expression of Sx1A-mEos2 and Sx1A^KARRAA^-mEos2.

### Fly stocks and rearing conditions

All flies were reared on standard yeast and sugar medium, and housed in plastic vials (Pathtech) at 25 °C. For the purposes of neuronal activation we expressed either the temperature-sensitive channel, dTRPA1 (ref. 37), or the light-sensitive channelrhodopsin, CsChrimson^40^, in the flies with constitutive Sx1A-mEos2 expression. C380-Gal4 was used to drive the expression of upstream activation sequence (UAS)-dTRPA1 and UAS-CsChrimson in motor neurons^36^. Expression of UAS-TeTx/LC in motor neurons was temporally controlled with a heat-sensitive Gal4 suppressor, Tub Gal80**ts** (refs 3, 49). We expressed TeTx/LC by raising the temperature to 32 °C for 2–4 h before imaging the filleted Sx1A-mEos2 larvae. We expressed Sx1A-mEos2 in the background of a temperature-sensitive NSF mutant (comt^ST17^) and used only the male larvae for imaging, as NSF comt^ST17^ is homozygous dominant on the X chromosome. We preheated larvae at 37 °C for 4 h before imaging to inactivate NSF comt^ST17^.

### Larvae dissection

Third instar *Drosophila* larvae of either gender, identified by their size, wandering behaviour and high motility were used for all dissections (except in optogenetic experiments where the females were exclusively used). Each larval dissection was done as previously described^63^ in Schneider’s Insect medium (Sigma, Life Sciences) and on a sylgard base (Sylgard 184 silicone elastomer kit, DOW Corning Corporation). Briefly, for each dissection the head and tail of the larva were held in place by minutien pins (Fine Science Tools). Iridectomy scissors (Fine Science Tools) were used to cut along the dorsal midline and then laterally so that the body wall could be pinned to form a flat hexagonal sheet. The intestinal and respiratory tracts were then detached to enable access to the abdominal wall muscles. In addition the larvae were de-cerebrated to prevent movement of the abdominal muscles during imaging. The whole preparation was deemed successful if the larva was still alive after the dissection (as evidenced by peristaltic contraction of the abdominal wall in response to noxious stimuli—for example, poking the ventral nerve cord with a minutien pin). All dissections were done using an optical stereo-microscope (SZ51, Olympus).

### Wide-field microscopy with oblique illumination

In optimizing the single-molecule imaging and localization of Sx1A-mEos2 *in vivo*, we used TIRF microscopy and oblique illumination. Dissected larvae pinned to a cylindrical sylgard base were inverted onto glass-bottomed culture dishes (*In Vitro* Scientific) filled with 2 ml Schneider’s Insect medium. NMJ synapses on the abdominal muscle 6 of the second segment were used for synaptic bouton imaging. A minimum of 12 NMJ chains were imaged from three or more different larvae for each condition. Localization and tracking of monomer Sx1A-mEos2 was achieved using the ELYRA PS.1 microscope (Zeiss), with a × 63 water-immersion objective (1.0 numerical aperture). Oblique TIRF illumination allowed visualization of the NMJ side embedded on the surface of the muscle. NMJs were located by illuminating Sx1A-mEos2 with a 488 nm laser. Single mEos2 molecules were visualized by photoconversion with a 405 nm laser and the single-molecule fluorescence of the resulting conversion excited with a 561 nm laser. Both lasers were used simultaneously and their power was adjusted to keep the number of the stochastically fluorescent molecules constant and spatially separated during the acquisition. The fluorescence was collected by a sensitive EMCCD (electron-multiplying charge-coupled device) camera (Evolve, Photometric). Zen Black acquisition software (2012 version; Carl Zeiss) was used in the recording and acquisition of movies during single particle tracking on the ELYRA microscope. During imaging, 15,000–25,000 frames were obtained from each NMJ preparation and images were captured at 33 Hz.

### Electrophysiology

Sharp intracellular recordings were made from third instar larvae as previously described^64^. In brief, larvae were dissected in ice-cold HL6 haemolymph-like solution (1.0 mM Ca^2+^ and 15 mM Mg^2+^) and were pinned to a glass dissection surface to gain access to the abdominal wall muscles. Intracellular electrodes (80–100 MΩ) were filled with 3 M potassium acetate and 3 M potassium chloride at a ratio of 2:1. Recordings were conducted at room temperature (25 °C) except in activation of dTRPA1-expressing larvae (30 °C) and TeTx/LC-expressing larvae (32 °C). Heating from room temperature to 30 °C was done via the continuous perfusion of HL6 that had slowly passed over a thermoelectric peltier heating device. Optogenetic stimulation of CsChrimson-expressing larvae was carried out using a red-orange light-emitting diode with a 617 nm wavelength^40^. Recordings of miniature endplate potentials were carried out in HL6 from muscle 6 on the second abdominal segment. Signals were amplified using an Axoclamp2B amplifier (Molecular Devices). Chart software (v.5.5.4) was used for acquisition. Recordings were processed in Axograph X (version 1.5.4, Axon Instruments) to obtain the frequency of depolarizations and the amplitude of evoked release. Excitatory junctional potentials recordings were stimulated at a frequency of 1 Hz. The quantal content was calculated by dividing the mean excitatory junctional potentials by the mean spontaneous end plate potentials.

### Cell culture and DNA transfection

PC12 cells were cultured and transfected as previously described^65^,^66^. In summary, cells were grown in Dubelcco’s modified Eagle’s medium (Gibco, Life Technologies) supplemented with 5% Serum Supreme (Gibco, Life Technologies), 5% heat-inactivated horse serum (Gibco, Life Technologies) and 0.5% GlutaMAX (Gibco, Life Technologies); the cells were maintained at 37 °C in 5% carbon dioxide. Sx1A-mEos2 plasmid transfection was done using Lipofectamine LTX and Plus Reagent (Invitrogen) as per the manufacturer’s instructions. Cells were replated onto poly-D-lysine-coated glass-bottomed culture dishes (MatTek Corporation) 24 h after transfection. Life-cell imaging was done on the third day after transfection.

### TIRF microscopy

Transfected PC12 cells were bathed in Buffer A (145 mM NaCl, 5 mM KCl, 1.2 mM Na_2_HPO_4_, 10 mM D-glucose and 20 mM HEPES, pH 7.4). The cells were then visualized using an inverted Roper Scientific TIRF microscope equipped with a perfect focus system and an ILas^2^ double laser illuminator (Roper Scientific). The microscope was fitted with a Nikon CFI Apo TIRF × 100 (1.49 numerical aperture) oil objective (Nikon Instruments) and an Evolve 512 delta EMCCD camera (Photometrics). Metamorph software was used for movie acquisition (Metamorph 7.7.8, Molecular Devices) at 50 Hz with 16,000 frames acquired for each cell kept at 37 °C. For PALM, a 405 nm laser was used to photoactivate the cells expressing Sx1A-mEos2, and a 561 nm laser was used for excitation of the resulting photo-converted single-molecule fluorescence signal. The sample was illuminated simultaneously with both the lasers. To isolate the mEos2 signal from auto-fluorescence and background signals, we used a double beam splitter (LF488/561-A-000, Semrock) and a double band emitter (FF01-523/610-25, Semrock, USA). To spatially distinguish and temporally separate the stochastically activated molecules during acquisition, the respective power of the lasers was adjusted (405 nm laser used 4–6% of initial laser power (200 mW) and 561 nm laser used 75–80% of initial laser power (200 mW)).

### Western blots

Adult flies heads were collected using dual sieves (#25 and #45, Thermo, Fisher, Australia). The heads were homogenized in extraction buffer (20 mM HEPES, pH 7.0, 100 mM potassium chloride, 5% glycerol, 10 mM EDTA, 0.1% TritonX-100 and cocktail of protease inhibitors (Roche)). Protein extraction was done using the Thermo Scientific Pierce BCA protein assay protocol (#23227). Quantified lysates were eluted in SDS sample buffer and boiled for 10 min. Proteins were loaded onto 12% SDS–PAGE gels. After separation, the proteins were transferred to a polyvinylidine fluoride membrane (Merck Millipore) in transfer buffer (25 mM Tris, 0.2 M glycine and 20% v/v methanol). Sx1A was detected using Sx1A antibody (mouse anti-8c3, #AB_528484 1:1,000; Developmental Studies Hybridoma Bank). To quantify VAMP2 cleavage by TeTx/LC, third instar larvae were used for protein extraction and detected with VAMP2 antibody (rabbit anti-VAMP2, #104 202 1:1000 Synaptic Systems). Mouse anti-β-actin (#ab6276, 1:1,000 Abcam) was used to detect actin. Uncropped scans are available in Supplementary Fig. 8.

### Data and statistical analysis

Fiji software (version 1.48d; Image J) was used for the management of files obtained during imaging (of larva motor nerve terminals, as well as PC12 cells) and was also used to convert the movies to the format recognizable for the image analysis software (from CZI to Tiff). A PALM-tracer plugin running on the Metamorph software was used to process the movies. The software localized fluorescent particles with sufficient luminous threshold in all movie frames and then followed the trajectories of each particle until the molecule photo-bleached or moved out of the field of view. Only tracks within eight frames or more were used for data analysis. The Metamorph software generated the MSD (μm^2^) using equation (1)^67^:


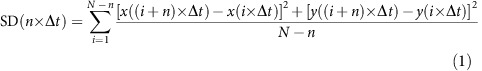


The diffusion coefficient *D* (μm^2^ s^−1^) was calculated for each trajectory, from linear fits of the first four points of the MSD versus time function using equation (2):


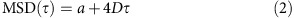


*N* is the number of data points in a trajectory, *a* is the offset constant that incorporates the effects of localization error and finite camera exposure, *Δt* is the time interval of each frame, *x* and *y* are the coordinates of a particle and *D* is the diffusion coefficient. The MSD was calculated for the time interval *τ=nΔt* for the entire duration of each trajectory. The first eight points of the MSD were averaged over all trajectories and plotted against time. The software also produced images of the tracks of each fluorescent molecule and its average intensity. Relative frequency distribution graphs and average MSD curves were obtained using Graph Prism (version 6.0). Trajectories with eight or more frames duration were considered for analysis of the MSD. The threshold for the mobile to immobile fraction was determined to be 0.021 μm^2^ s^−1^ as previously described^38^. The robust regression and outlier removal (ROUT) outlier test on Graph Pad Prism was used to identify and remove outliers. Data are presented as mean±s.e.m. and comparisons were done with Student’s *t*-test (two-tailed distribution, unpaired) unless otherwise stated.

### Single-molecule localization microscopy and cluster analysis

Sx1A-mEos2-expressing larvae were fixed with 4% paraformaldehyde diluted in phosphate buffered saline (PBS) for 30 min before imaging and then blocked for 30 min (PBS with 0.1% TritonX-100, and 3% normal goat serum, Sigma). Mouse primary antibody Brp^Nc82^ (#AB_2314866, Developmental Studies Hybridoma Bank) was used to stain active zone protein (bruchpilot) through overnight incubation at 4 °C (1:100) in blocking buffer. Following extensive washes in PBS, Alexa Fluor 647-conjugated goat anti-mouse antibody was used as the secondary antibody (#A-21236 1:500, Molecular Probes). Tetraspeck beads were then added at 1/500 in PBS for 15 min. Images were acquired on an ELYRA PS1 microscope equipped with a × 100 objective (α Plan-Apochromat × 100/1.46 oil immersion) and an EMCCD camera. Sx1A-mEos2 molecules were simultaneously photo-converted with a 405 nm laser and excited using a 561 nm laser. A total of 15,000 frames were acquired at a rate of 33 Hz.

The acquired time-lapse movies were processed to retrieve coordinates for the individual molecules using Zen software (Zeiss). SML images were *xy*-drift-corrected using Zen’s automated fiducial markers and Zen’s affine transform algorithms. To partially account for multiple appearances of molecules, localizations that appeared within 1 frame and 1 pixel of one another were consolidated. The data sets were reconstructed with a pixel size of 10 nm and regions of interest were selected from reconstructed two-dimensional histograms. A custom-written programme^68^ in Matlab (The Mathworks, 2014) quantified the clustering of the proteins using an auto-correlation function, which provides information on the likelihood of finding a molecule at a given distance away from another molecule^43^,^69^. The calculated auto-correlation (*g*(*r*)) values were fitted to equation (3) to obtain the characteristics of the clusters:


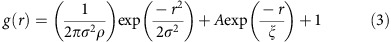


*ρ* is the density of the image, *σ* is the s.d. of the point spread function and *ξ* is the correlation length (that is the average radius of the clusters). The resolution of the image was calculated as *σ*/√2. *Α* is the amplitude of the exponential decay function. The average number of molecules per cluster was approximated by equation (4):


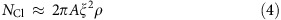


*A*, in equation 4 is a constant that has been approximated as the amplitude of the protein auto-correlation function extrapolated to *r*=0.

### Hidden Markov modelling

We used vbSPT analysis to infer the number of hidden diffusive states from Sx1A trajectories as previously described^39^. We allowed a maximum of five hidden states, which is more than the initially expected number of hidden states^39^. We analysed NMJ chains with a minimum of ∼1,000 trajectories. The average number of trajectories per NMJ chain was ∼3,500 and the average trajectory length was ∼11 time steps. In the majority of NMJ chains, a three-state model was the best fit, with each state representing immobile, slow mobile and fast mobile diffusive states. In a few other NMJ chains, a two-, four-state or five-state models provided the best fit. The apparent diffusion coefficients of the additional states in the four- or five-state models were similar to that of either the slow mobile or fast mobile diffusive states, suggesting degenerate diffusive states as previously observed^70^. We therefore analysed the parameters of the three-state model in Fig. 3 and Supplementary Fig. 4. In addition, we analysed the best-fit models by pooling the different diffusive states (two, three, four or five states) into immobile, slow mobile and fast mobile states based on the diffusion coefficients. We again found that the immobile state occupancy decreased (*P*<0.05, Mann–Whitney *U*-test), the fast mobile state occupancy increased (*P*<0.01) and the slow mobile state did not change (*P*>0.05) upon dTRPA1 expression at 30 °C. There was no significant change in any state occupancies upon dTRPA1 expression at 25 °C (*P*>0.05).

### Data availability

All data used in this study can be made available on request to the corresponding author.

## Additional information

**How to cite this article:** Bademosi, A. T. *et al. In vivo* single-molecule imaging of syntaxin1A reveals polyphosphoinositide- and activity-dependent trapping in presynaptic nanoclusters. *Nat. Commun.* **7,** 13660 doi: 10.1038/ncomms13660 (2017).

**Publisher's note:** Springer Nature remains neutral with regard to jurisdictional claims in published maps and institutional affiliations.

## Supplementary Material

Supplementary Information Supplementary Figures 1-8 and Supplementary Table 1

Supplementary Movie 1 Sx1A-mEos2 single molecule imaging at the motor nerve terminal. sptPALM imaging of Sx1A-mEos2 on a type-I bouton of abdominal muscle 6. Movie acquired at 33Hz and played back at acquired rate. Scale bar 5μm.
