# Erratum: *In vivo* single-molecule imaging of syntaxin1A reveals polyphosphoinositide- and activity-dependent trapping in presynaptic nanoclusters

**DOI:** 10.1038/ncomms14492

**Published:** 2017-02-09

**Authors:** Adekunle T. Bademosi, Elsa Lauwers, Pranesh Padmanabhan, Lorenzo Odierna, Ye Jin Chai, Andreas Papadopulos, Geoffrey J. Goodhill, Patrik Verstreken, Bruno van Swinderen, Frédéric A Meunier

Nature Communications 7: Article number: 13660; DOI: 10.1038/ncomms13660 (2016); Published: 12 16 2016; Updated: 02 09 2017

This Article was originally published with an incorrect publication date. The paper was due to be published on 3 Jan 2017, but due to an error was published earlier on the 16 Dec 2016. The publication date in both the PDF and HTML versions of the paper has been updated to reflect this.

