# Publisher Correction: *In vivo* single-molecule imaging of syntaxin1A reveals polyphosphoinositide- and activity-dependent trapping in presynaptic nanoclusters

**DOI:** 10.1038/ncomms16191

**Published:** 2018-04-10

**Authors:** Adekunle T. Bademosi, Elsa Lauwers, Pranesh Padmanabhan, Lorenzo Odierna, Ye Jin Chai, Andreas Papadopulos, Geoffrey J. Goodhill, Patrik Verstreken, Bruno van Swinderen, Frédéric A. Meunier

Nature Communications 7: Article number: 13660; DOI: 10.1038/ncomms13660 (2016); Published: 12 16 2016; Updated: 04 10 2018

The original HTML version of this Article had an incorrect volume number of 8; it should have been 7. This has now been corrected in the HTML; the PDF version of the Article was correct from the time of publication.

