# Supplementary material for: In vivo single-molecule imaging of syntaxin1A reveals polyphosphoinositide- and activity-dependent trapping in presynaptic nanoclusters
Source: Nat Commun. 2016 Dec 16;7:13660. doi: 10.1038/ncomms13660 (PMC5171881; doi:10.1038/ncomms13660)
Supplement: Supplementary Information — Supplementary Figures 1-8 and Supplementary Table 1 [file ncomms13660-s1.pdf]

Supplementary Figure 1

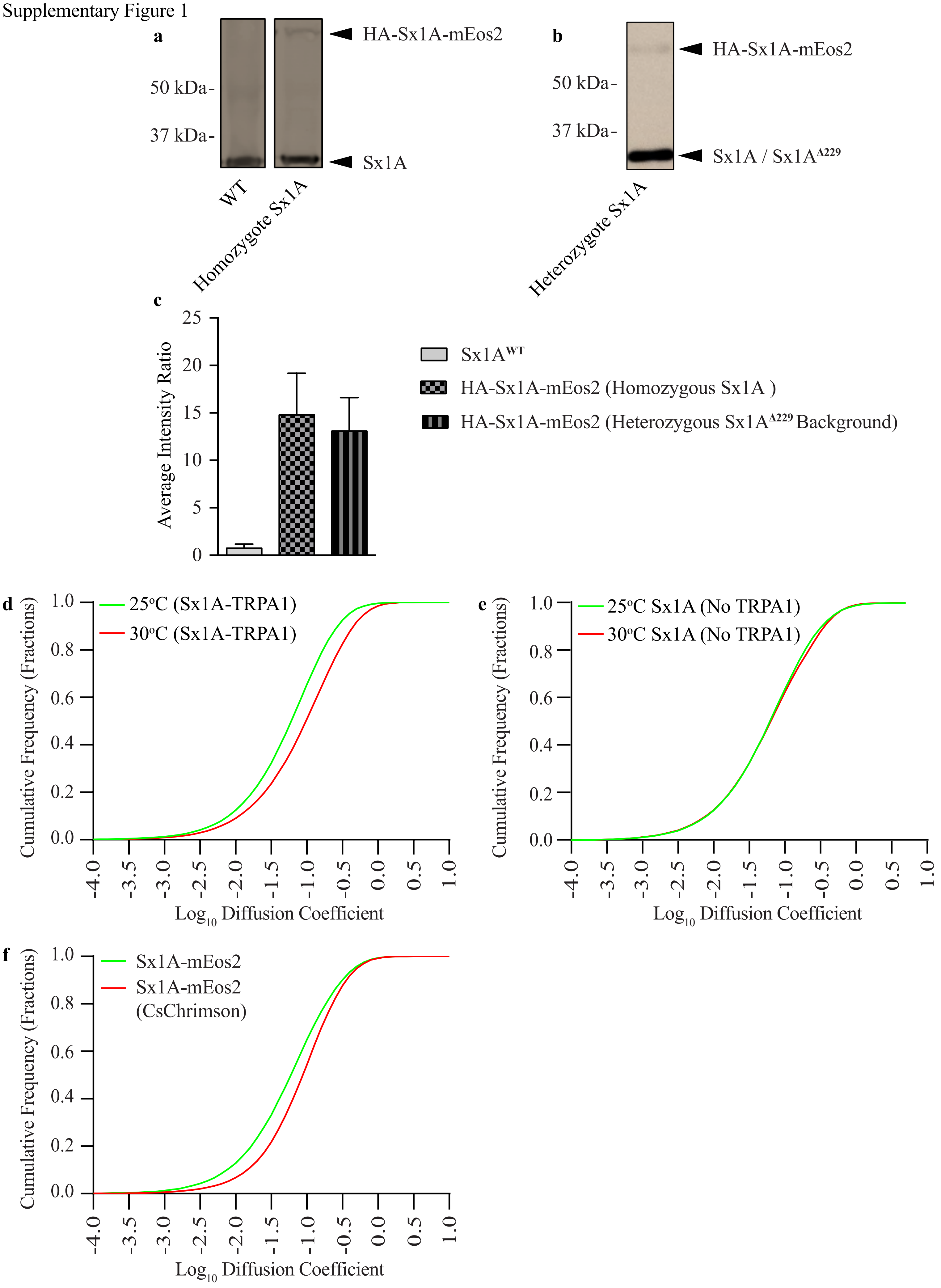

Supplementary Figure 1. Sx1A-mEos2 expression and cumulative frequency distribution. (a-c) Sx1A-mEos2 expression was analyzed by western blot in larvae with full endogenous Sx1A expression and in larvae with heterozygous expression of Sx1A^{Δ229}. Sx1A-mEos2 expression was much lower when compared to endogenous Sx1A. Average intensity measurement showed that Sx1A-mEos2 expression was also low in the heterozygous null Sx1A mutant (14.8±4.0 to 13.1±3.5). (d) Cumulative frequency distribution of diffusion coefficients of Sx1A-mEos2 upon dTRPA1 temperature activation from 25°C to 30°C; $P<0.0001$, Kolmogorov-Smirnov test. (e) Cumulative frequency distribution of diffusion coefficients showed temperature elevation from 25°C to 30°C does not alter Sx1A -mEos2 mobility; $P>0.05$, Kolmogorov-Smirnov test. (f) Cumulative frequency distribution of diffusion coefficient of Sx1A-mEos2 upon CsChrimson-retinal-mediated synaptic transmission showed increased mobility; $P<0.0001$, Kolmogorov-Smirnov test. Mean±SEM are plotted.

Supplementary Figure 2

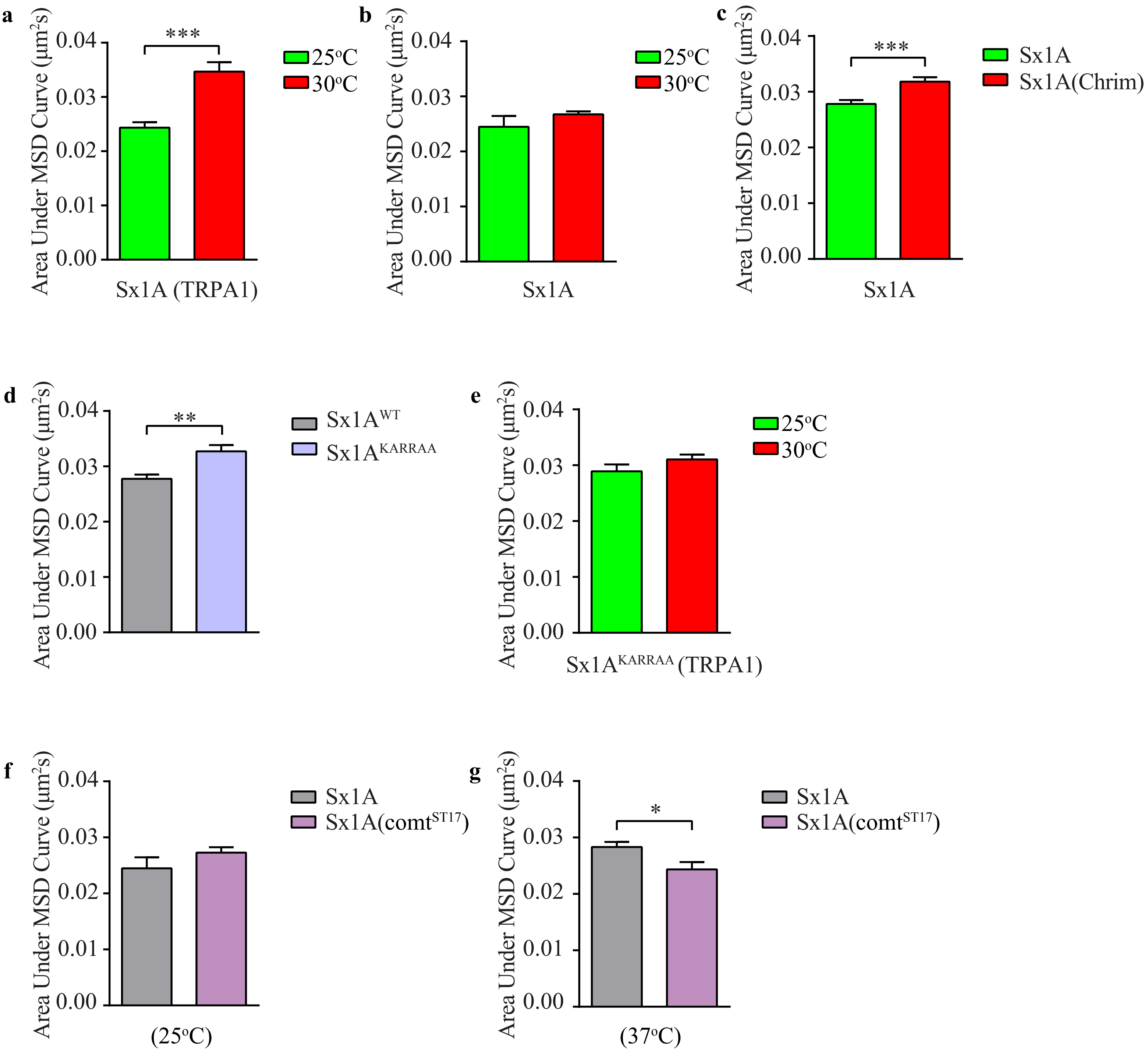

Supplementary Figure 2. Area under the mean square displacement curves. (a-g) Shows the area under the MSD curve for Sx1A mobility in each of the different conditions. Statistical tests were performed using the student's t test (two-tailed distribution, unpaired). *P<0.05, **, P<0.01, ***, P<0.001. Mean±SEM are plotted.

Supplementary Figure 3

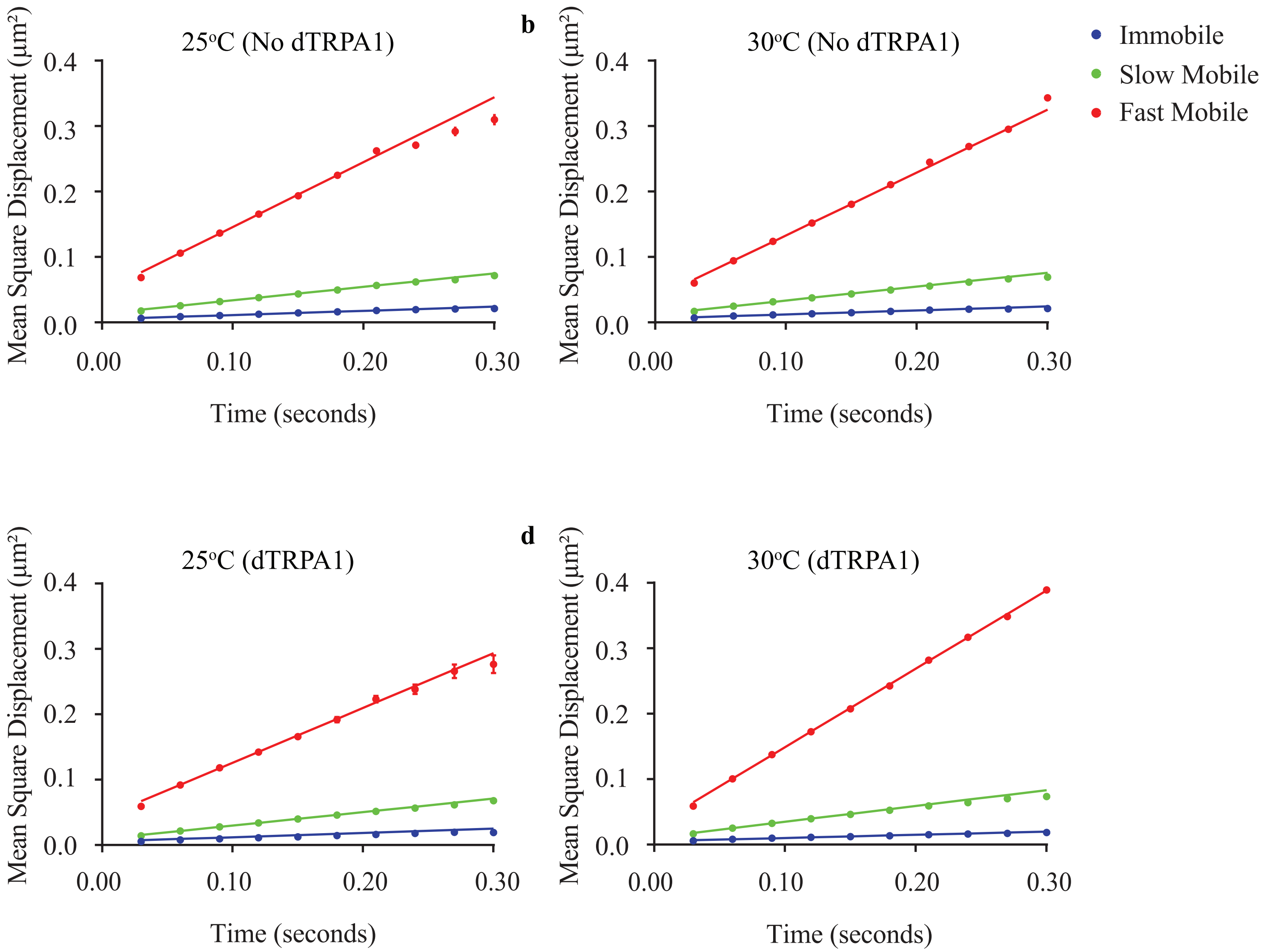

Supplementary Figure 3. Mean square displacement analysis of each diffusive state. Mean square displacement of trajectories (symbols) that did not undergo stochastic switching from (a) NMJ chains without dTRPA1 expression at 25ºC (n = 2138 (blue), 4140 (green), 8927 (red) trajectories, 11 NMJ chains), (b) NMJ chains without dTRPA1 expression at 30ºC (n = 1932 (blue), 4475 (green), 3867 (red) trajectories, 10 NMJ chains), (c) NMJ chains with dTRPA1 expression at 25ºC (n = 2635 (blue), 4214 (green), 2341 (red) trajectories, 12 NMJ chains), and (d) NMJ chains with dTRPA1 expression at 30ºC (n = 2695 (blue), 7994 (green), 19000 (red) trajectories, 11 NMJ chains). Error bars indicate SEM. Solid lines are a linear fit to the second, third and fourth time lags.

Supplementary Figure 4

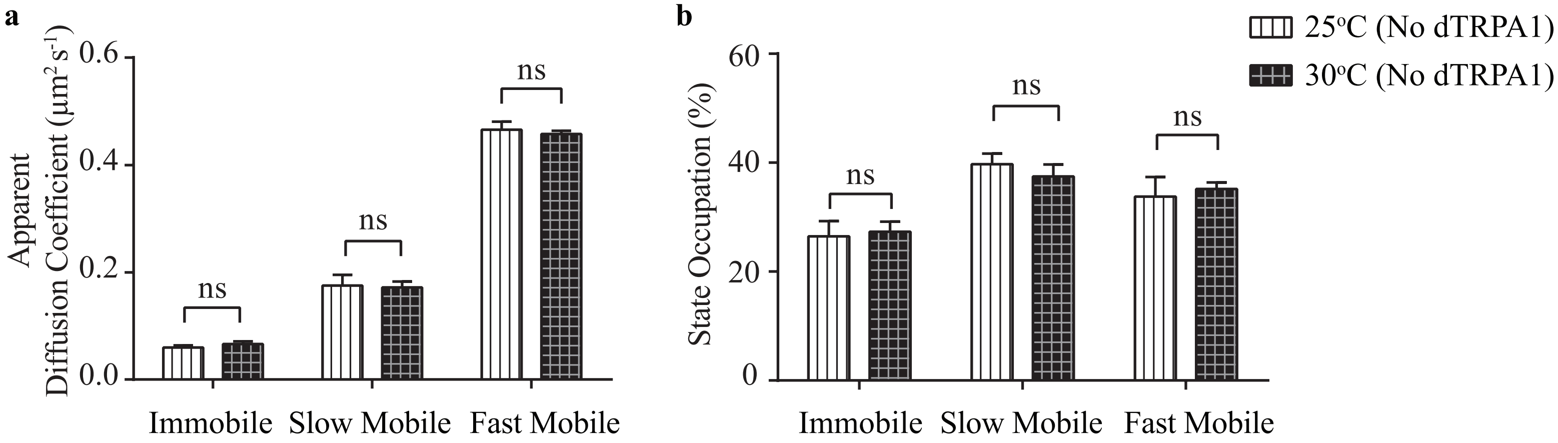

Supplementary Figure 4. Temperature elevation did not affect hidden Markov model parameters in the absence of dTRPA1 expression. (a) Apparent diffusion coefficients and (b) state occupations inferred by analysing trajectories from NMJ chains without expression of dTRPA1 at 25ºC and 30ºC. Statistical tests were performed using Mann-Whitney U test (ns indicates $P>0.05$). Mean±SEM are plotted.

Supplementary Figure 5

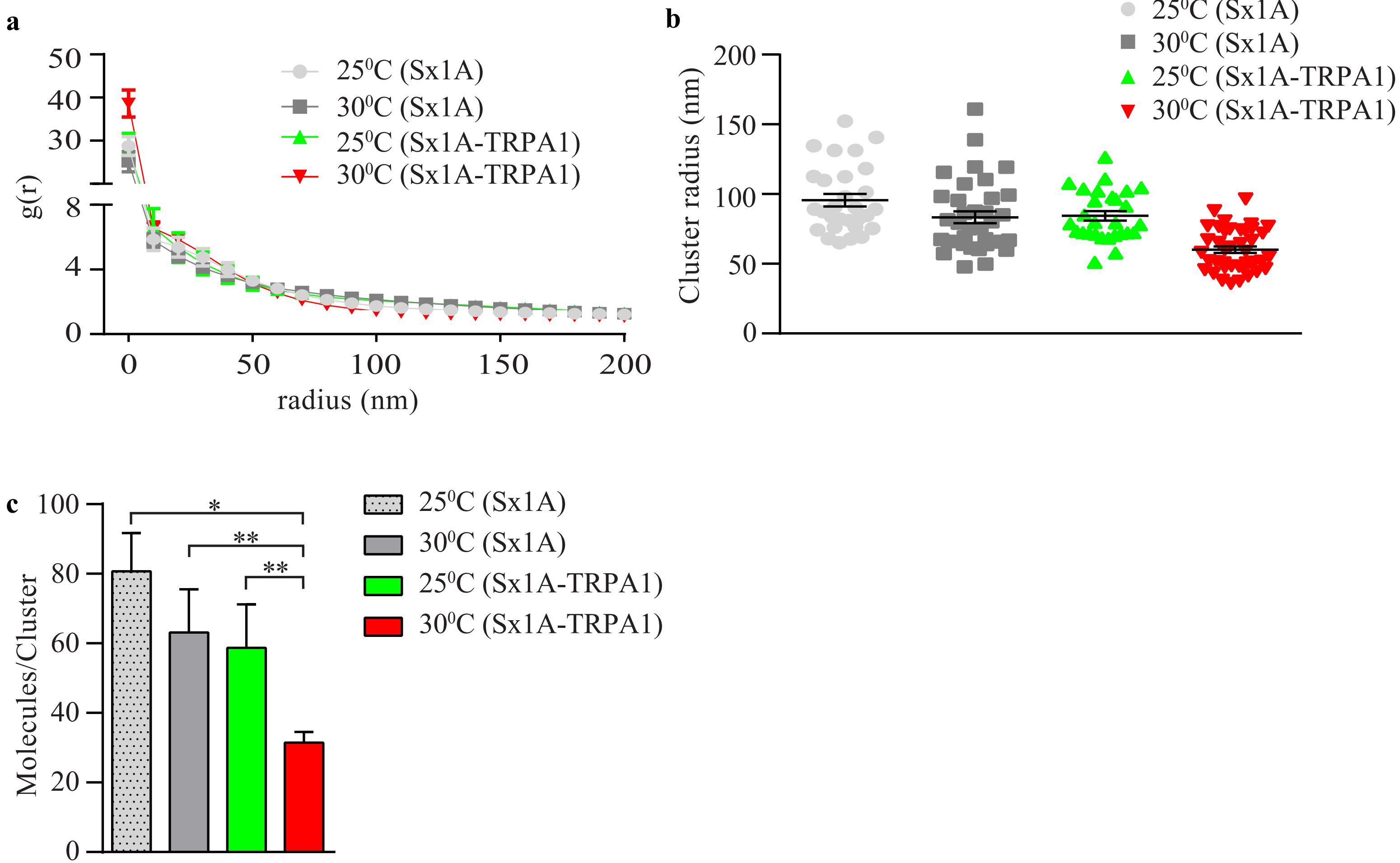

Supplementary Figure 5. Thermogenetic-mediated increase in presynaptic transmission decreased Sx1A-mEos2 molecules per cluster. (a) Average of auto-correlation functions of Sx1A-mEos2 (in the presence or absence of dTRPA1 expression) upon temperature increase from 25°C to 30°C. (b) Cluster radius scatter plot distribution of Sx1A-mEos2 upon elevation of presynaptic activity. (c) The average number of molecules per cluster of Sx1A-mEos2 significantly decreased from 58.7±12.5 to 31.4±3.6 with a dTRPA1-mediated increase in synaptic activity. Temperature did not significantly alter the Sx1A-mEos2 molecules per cluster (25°C, 80.7±10.9 and 30°C, 63.1±12.4). Statistical tests were performed using student's t test (two-tailed distribution, unpaired). *, $P<0.05$, **, $P<0.01$, ***, $P<0.001$. Mean±SEM are plotted.

Supplementary Figure 6

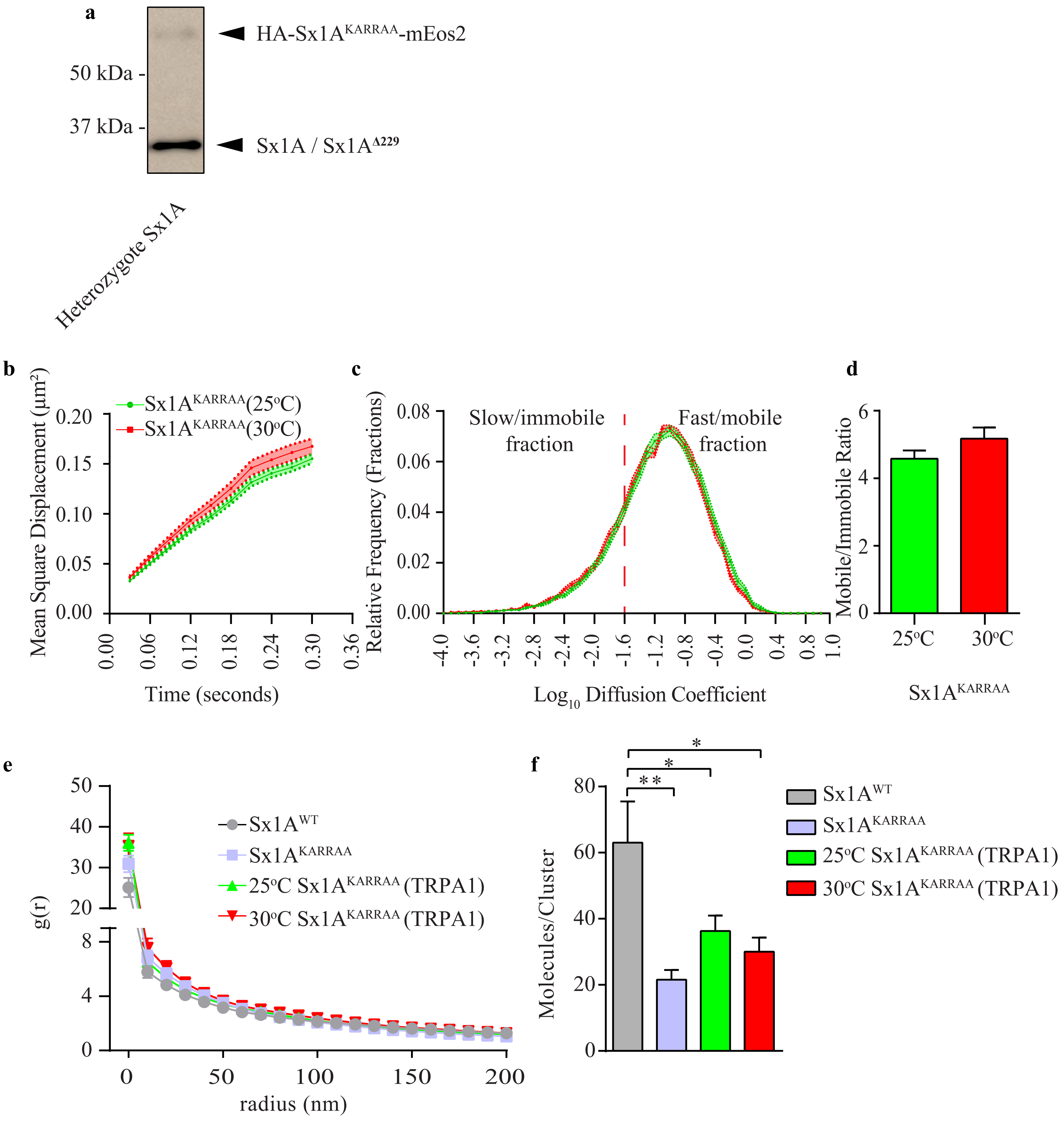

Supplementary Figure 6. $Sx1A^{KARRAA}$-mEos2 expression, mobility and molecules per cluster.
(a) $Sx1A^{KARRAA}$-mEos2 expressed in the Sx1A-null mutant background with heterozygous expression.(b-d) MSD and diffusion coefficient distribution. Note that a change in temperature from 25°C to 30°C did not alter $Sx1A^{KARRAA}$-mEos2 mobility. The mobile to immobile ratio was also not altered (25°C, 4.6±0.2 to 30°C, 5.2±0.3, n=15 NMJ chains for $Sx1A^{KARRAA}$-mEos2 at 25°C and 17 NMJ chains for $Sx1A^{KARRAA}$-mEos2 at 30°C; average of ~1,600 trajectories per NMJ chain). (e) Average of auto-correlation functions of $Sx1A^{KARRAA}$-mEos2 upon a dTRPA1-mediated increase in synaptic activity. (f) The average number of molecules per cluster of $Sx1A^{KARRAA}$-mEos2 was significantly lower (21.5±2.9) compared with Sx1A-mEos2 (63.1±12.4). The thermogenetic-mediated increase in synaptic activity did not alter the average number of $Sx1A^{KARRAA}$-mEos2 molecules per cluster (36.3±4.7 at 25°C to 30.0±4.2 at 30°C). Statistical tests were performed using student's t test (two-tailed distribution, unpaired). *, $P<0.05$, **, $P<0.01$, ***, $P<0.001$. Mean±SEM are plotted.

Supplementary Figure 7

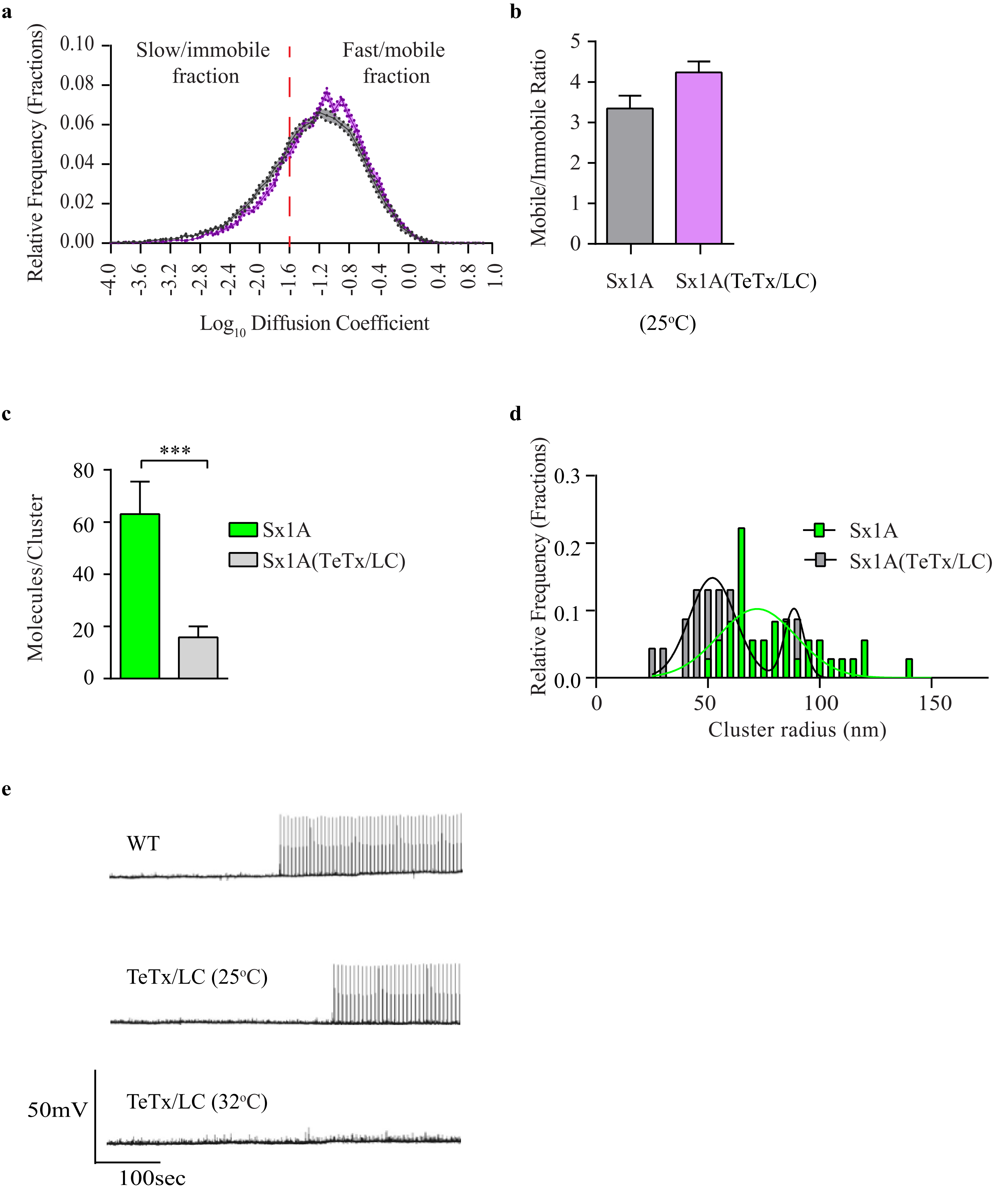

Supplementary Figure 7. Change in Sx1A-mEos2 molecules per cluster and cluster size distribution upon TeTx/LC expression. (a-b) The diffusion coefficient distribution of Sx1A-mEos2 was not altered 25°C when tubulin Gal80 suppressed the expression of TeTx/LC. The mobile to immobile ratio of Sx1A-mEos2 was 3.3±0.3 and 4.2±0.3 with TeTx/LC suppressed (n=15 NMJ chains for Sx1A-mEos2 only and 10 NMJ chains for Sx1A-mEos2 with TeTx/LC suppressed; average of ~1,900 trajectories per NMJ chain). (c) TeTx/LC expression significantly decreased the average number of molecules per Sx1A-mEos2 cluster from 63.1±12.4 to 15.8±4.2. Statistical tests were performed using the Mann-Whitney U test ***P<0.001. (d) Cluster size peak distribution for Sx1A-mEos2 was 72.0±5.40 and changed to a two peak distribution at 52.0±0.9 and 88.5±0.9 upon TeTx/LC expression. (f) Excitatory junction potentials in wild-type Drosophila larvae, Sx1A-mEos2 transgenic larvae with tubulin Gal80 suppression of TeTx/LC (25°C) and Sx1A-mEos2 larvae with TeTx/LC expression (32°C). Mean±SEM are plotted.

Supplementary Figure 8

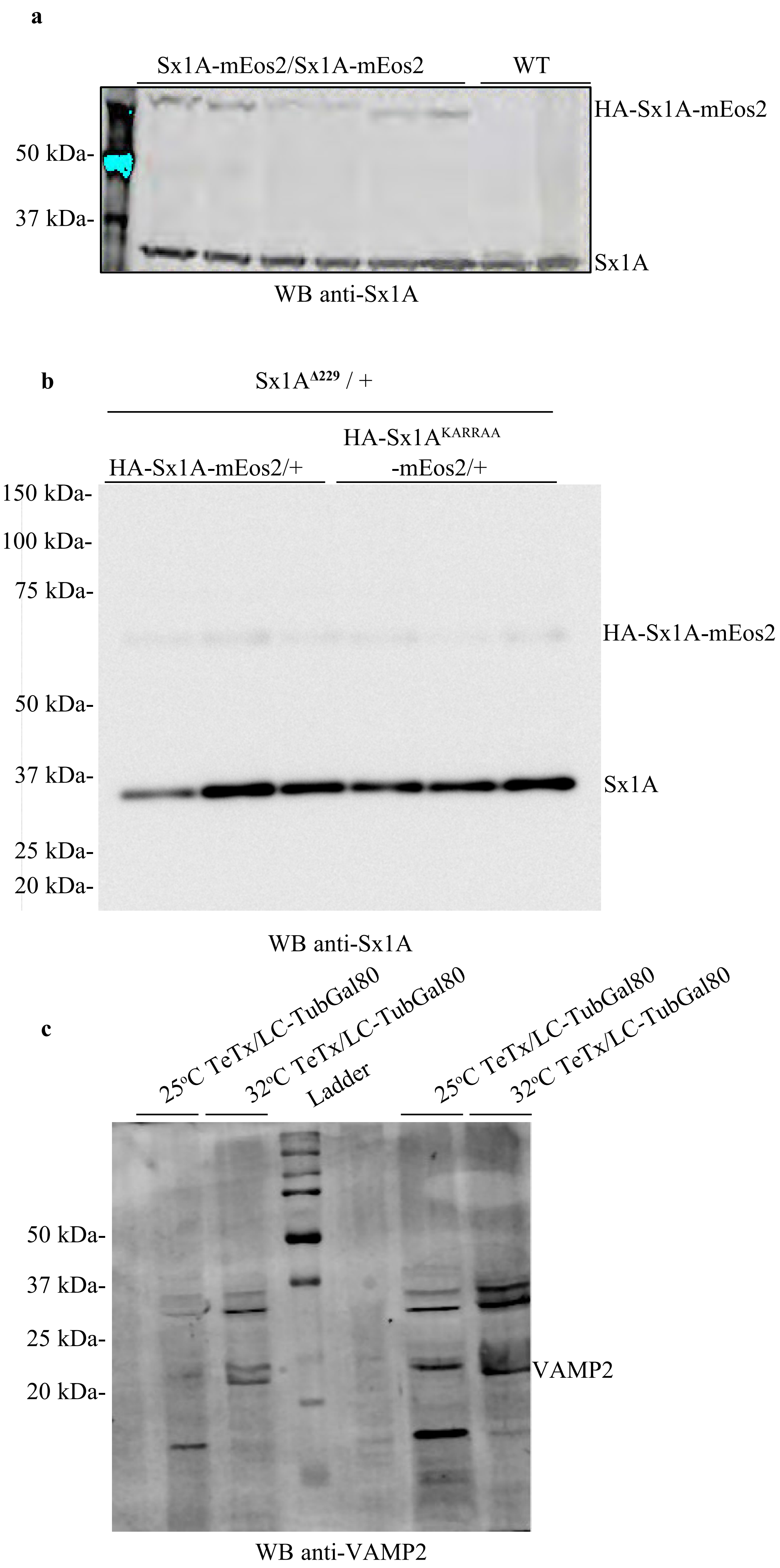

Supplementary Figure 8. Uncropped scans of western blots. (a) Sx1A-mEos2 expression was analysed by western blot in larvae with full endogenous Sx1A expression. (b) Sx1A-mEos2 and Sx1A^{KARRAA}-mEos2 expression was analysed by western blot in larvae with heterozygous expression of Sx1A$^{\Delta 229}$. (c) Cleavage of VAMP2 by temperature dependent expression of TeTx/LC was anlaysed by western blot.

Supplementary Table 1. Primers used in making Sx1A-mEos2 and Sx1A^{KARRAA}-mEos2 construct. They include the forward and reverse sequences for Sx1A and mEos2, mEos2 check, as well as the sequence for the amino acid linking Sx1A to mEos2.

**Primer**	**Sequence**
F-Sx1A-mEos2	GGGCATCTTAGCGGCCTCATATGTTAGCAGTTATTTCATGATGAGTGCGATTAAGCCAG
R-Sx1A-mEos2	GGAGTGAATGTGGCGTGTAAGTAATTGCATTCGAGATTAATCGTCTGGCATTGTCAGGC
F-mEos2 Genomic	TGCTGTTGCTCATTCTGGATTGCCTGACAATGCCAGACGATAAATCTCGAATGCAATTAC
R-mEos2 Genomic	GTGAGAAATGAAATCATAGCTGAAACCGAAATCCATTAATTTCTGTTAACTTTTCTGGC
F-mEos2 Check	TGAAGGAAATGCCCATTACC
R-mEos2 Check	GAGACTCTTGCTTACTAACC
12 Amino acid Linker	GAG GTA CCG CGG GCC CGG GAT CCA CCG GTC GCC ACC

!
